# Optical and Electron Transparent Polycrystalline Boron Doped Diamond Membranes for Nanoscale Correlative Structure-Electrochemical Measurements

**DOI:** 10.1021/acsnano.5c13873

**Published:** 2025-12-03

**Authors:** Pei Zhao, Daniel Houghton, Joshua J. Tully, Dimitrios Valavanis, Patrick R. Unwin, Richard Beanland, Yisong Han, Marc Walker, Mark E. Newton, Julie V. Macpherson

**Affiliations:** † Department of Chemistry, 2707 University of Warwick, Coventry CV4 7AL, U.K.; ‡ Department of Physics, University of Warwick, Coventry CV4 7AL, U.K.

**Keywords:** boron doped diamond (BDD), scanning transmission electron microscopy (STEM), diamond membranes, scanning electrochemical cell microscopy (SECCM), gold electrodeposition, nanoparticles

## Abstract

The ability to synthesize and structurally interrogate tailored nanostructured materials is a major theme in nanoscience. It requires multimodal methods and platforms, with high resolution microscopy being a central technique. Here a nanofabrication procedure is demonstrated to enable the production of free-standing, electron and optically transparent conductive polycrystalline boron doped diamond (BDD) membranes. These serve as both electrodes onto which nanomaterials can be produced using electrodeposition strategies and platforms for correlative microscopy. The membrane fabrication method involves ion implantation into free-standing BDD to create a damage layer ca. a micron below the surface, high-temperature annealing, electrochemical etching to lift-off the ca. micron thick layer of BDD and optimized reactive ion etching to thin the layer down. The methodology results in ∼50 nm thin BDD membranes of area > 5 mm^2^ with low surface roughness (∼1.7 nm RMS) comprising grains, tens of μm in size. Going thinner, and producing larger areas, is also possible using this approach. Electron diffraction of the BDD membrane reveals a dominant (110) crystallographic orientation and annular dark field (ADF) scanning TEM (STEM) shows atom resolution of supported metallic (gold) nanostructures and isolated single atoms is achievable. Experimental optical transmissions > 50% are demonstrated. The performance of the BDD membrane for correlative microscopies is assessed against the commonly used carbon film TEM substrate and shown to be superior. Finally, using combined scanning electrochemical cell microscopy and ADF-STEM, the impact of electrode potential on the size, number density and crystal quality of electrodeposited gold nanoparticles on the BDD membrane is examined. The pivotal role electrochemical deposition potential plays in controlling nanoparticle crystal quality i.e. defect free single crystal versus polycrystalline NP is demonstrated.

## Graphical abstract

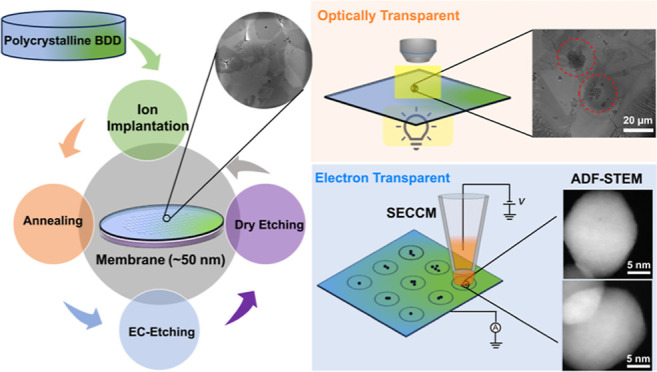

## Introduction

Metal nanostructures including isolated atoms, atom clusters and nanoparticles (NP) play a significant role in clean and sustainable energy conversion and storage systems.^1^ Noble metals, such as platinum, palladium, iridium, and gold, are widely used due to their enhanced electrocatalytic activity toward key redox processes associated with e.g. fuel cells, water splitting and sustainable fuel synthesis.^2–5^ These metals are used in NP form due to their high surface area, tunable activity and selectivity,^6,7^ as well as deployment in high mass transport regimes. More recently, supported single atoms and atom clusters have also shown excellent promise for these applications.^8,9^ Atom to nanosized structures, on the electrode surface, can be formed a variety of ways including, for example, chemical synthesis followed by deposition,^10^ or direct electro- (or photo-) deposition.^11,12^

Size, crystallography and crystalline quality are known to influence activity, selectivity and the stability of the NPs;^7,13–15^ although the factors which influence atom cluster activity are less clear.^16,17^ Identification and structural evaluation of nanostructures are commonly undertaken using transmission electron microscopy (TEM) and related techniques. For investigations into the effect of electrode potential (or current) on the formation or electrocatalytic response of individual nanostructures, it is beneficial that the TEM support can also function as the electrode. This requires the support to be electrically conductive, provide electrochemical stability in the potential region of interest and have no electrochemically interfering responses. Such supports then enable fundamental questions to be addressed, at the single atom level, for example, how does nanostructure: (i) influence electrocatalytic activity or; (ii) change in response to long-term electrocatalysis?^18^ (iii) How do electrodeposition parameters impact nanostructure morphology?

Common TEM substrates for combined electrochemical-TEM, where imaging takes place ex-situ, typically consist of a metal mesh (e.g., gold or copper) covered by a film of predominantly sp^2^ carbon.^19^ This is thin enough (10–30 nm) for transmission of the electron beam and high resolution imaging of supported nanostructures. This approach also offers isolated single atom resolution. For identical location electrochemical TEM, where the substrate is imaged, then placed in solution for electrochemical treatment, removed and then imaged again in the same location, the TEM substrates are labeled with index grids containing symbols or coding to aid location identification.^20^ For in situ electrochemical TEM, specialized microfabricated cells containing integrated thin film metal or carbon electrodes are adopted enabling combined TEM-electrochemical measurement in the electrolyte. However, this methodology suffers from both reduced resolution, compared to vacuum measurements, and issues associated with the stability of the analyte/electrolyte, due to the electron beam passing through the solution.^21^

While the substrates discussed above have advanced combined electrochemical–TEM measurements, there are inherent limitations that need to be overcome. For example, when working at highly oxidative potentials associated with e.g. the oxygen evolution reaction, both carbon and metal films can corrode.^22,23^ Metal films are, furthermore, not ideal supports for studying the isolated electrochemical responses of metal NPs. Thin carbon films are also fragile and can be easily damaged when handling.^24^ By contrast, boron doped diamond (BDD), is resistant to corrosion in the oxygen evolution region,^19^ is mechanically stronger than the carbon used in TEM grids or that produced via pyrolysis,^25^ is chemically robust, is an excellent thermal conductor and can be used free from a metal support.^26^ Initial work thinned BDD to electron beam transparency^27,28^ using an argon ion milling/polishing process, commonly employed in TEM sample preparation. However, as this process heterogeneously thinned the surface, only a region a few microns in size^19^ across the entire BDD surface (3 mm disk) was thin enough to attain electron beam transparency. To maximize the potential of BDD as an electron transparent electrode it is necessary to develop nanofabrication procedures that enable production of <100 nm thickness free-standing BDD membranes over much larger length scales (∼ mm to cm). Such membrane electrodes are expected to also show high optical transparency.

Scanning electrochemical cell microscopy (SECCM) uses an electrolyte-filled pipette of micro to nano dimensions to create a micro-nano sized electrochemical cell with an appropriate substrate. In this way electrochemical processes can be mapped on the micro-nano scale and many measurements are made on the same platform by simple movement of the pipette to a new location, ultimately enabling combinatorial approaches. SECCM has been employed with a variety of thin film (semi-transparent) platforms to provide correlative electrochemical and optical/electron microscopy measurements and enhance understanding of the system of interest. Such substrates range from graphene,^29^ conventional carbon film TEM grids^30,31^ and sputtered carbon on silicon wafers,^32^ to pyrolyzed carbon on quartz slides^33^ and TEM silicon nitride windows.^25^

In this work, a new fabrication procedure is described which enables the production of large area (>5 mm^2^), thin, large grain size polycrystalline BDD membranes, using an ion implantation, annealing, lift-off and etch methodology. The transparency of the resulting membrane to both light and electrons is demonstrated. Their dual functionality is illustrated via an investigation of the impact of electrode potential on gold electrodeposition nanostructure morphology and crystallinity using SECCM and annular dark field (ADF) scanning TEM (STEM). SECCM is employed to systematically vary the applied electrode potential, as a function of tip position on the BDD membrane, with the resulting nanostructures imaged using ADF-STEM.

## Results and Discussion

### Diamond Membrane Production

Free-standing oxygen-terminated polycrystalline BDD, doped in all regions, above the metallic threshold (>10^20^ boron atoms cm^–3^) and containing minimal sp^2^ carbon, was used herein.^34^ The BDD was grown thick enough (>0.4 mm) such that it could be removed from the non-diamond growth substrate and the top surface polished, using a resin-bonded wheel, to low surface roughness ∼ nm RMS. Grain sizes were typically tens of μm, significantly larger than BDD grown in thin film form which remains attached to a growth substrate.^26^ The procedure to create free-standing electron transparent BDD membranes from this starting material includes five sequential steps, shown in Figure 1. The presence of larger grains in the BDD was important as they help maintain the structural integrity of the thin BDD membranes during lift-off and subsequent use (vide infra).

**Figure 1:**
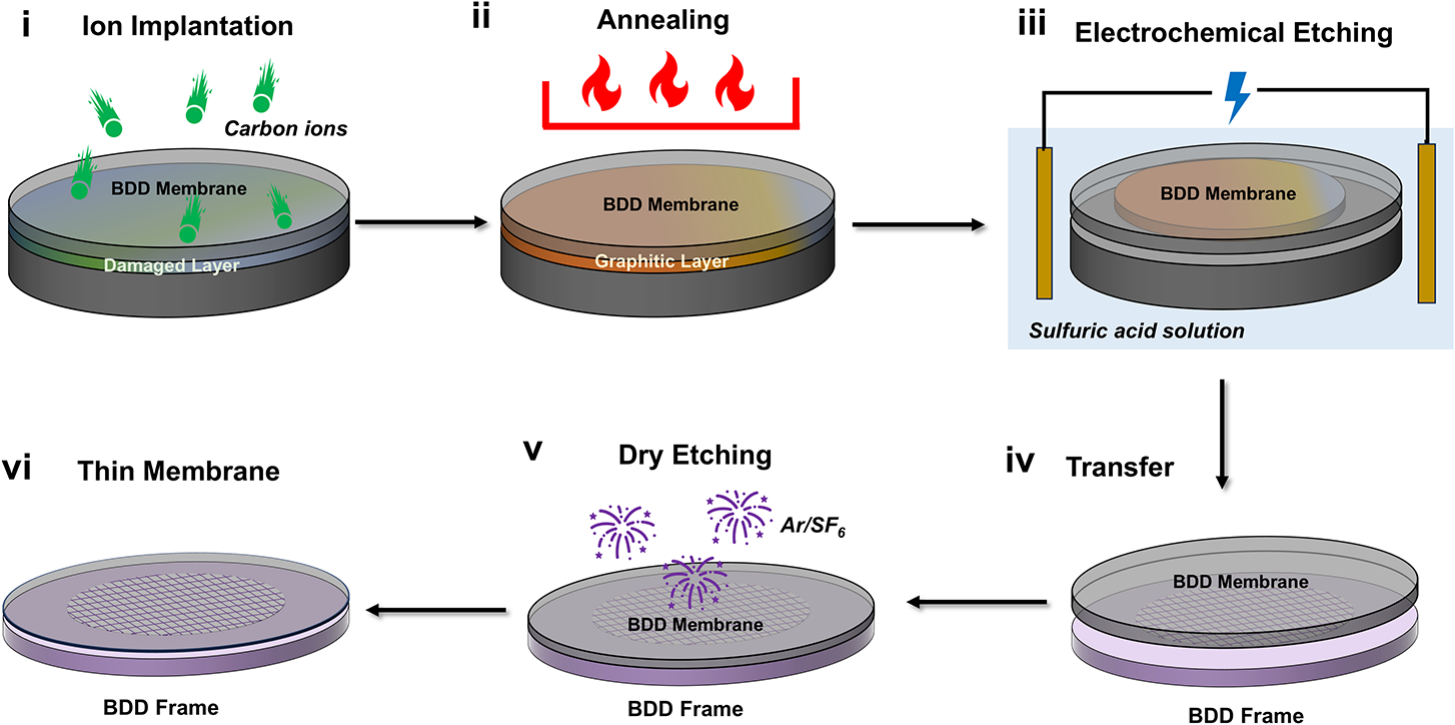
Illustration of the BDD electron beam transparent electrode fabrication process. (i) The ion implanted damage layer (green layer) is (ii) graphitized during the anneal process (orange layer) and subsequently (iii) electrochemically etched away (receding orange layer) to reveal the BDD membrane. (iv) The membrane is transferred to a support structure and (v) dry etched to thin down to electron transparency to reveal the final (vi) thin membrane. Note that the drawing is not to scale.

Step (i): implantation of 2 MeV carbon ions to create a sub-surface damage layer in the BDD, this layer begins ∼ 0.9 μm below the surface and is estimated to be ∼ 0.4 μm thick; Supporting Information Section 1.^35,36^ (ii) High temperature annealing (1300 °C) to convert the ion implanted region to graphitic carbon. (iii) Electrochemical etching of the graphitic region to “lift-off” the ∼ 0.9 μm thick BDD membrane.^36^ (iv) Transfer of the membrane to a supporting (mesh) structure, described in detail in Supporting Information Section 2 and (v) inductively coupled plasma reactive ion etching (ICP-RIE) dry etching of the membrane, using Ar/SF_6_, to thin the entire membrane to electron beam transparency (<100 nm), Supporting Information Section 3. Ar/SF_6_ was chosen as the RIE etchant, given the moderate BDD etch rate of 87 ± 3 nm/min (Supporting Information Section 3) and low resulting surface roughness, compared to the other RIE options assessed in Supporting Information Section 3. Specific details on steps (i–iii) are described in the Experimental Section. Similar procedures (i–iii) have been used to create single crystal diamond membranes for photonic and quantum device applications.^37–39^ However, to the best of our knowledge, there is no literature describing fabrication of polycrystalline diamond or BDD membranes using this procedure. Compared to single crystal BDD, polycrystalline BDD currently offers a reduced cost and ability to grow easily at scale. Steps (iv) and (v) have been specifically developed to enable the production of supported conductive BDD membranes optimized for correlative TEM and SECCM measurements.

### Characterization of the BDD TEM Membrane

An optical image of a typical lifted-off, 3 mm diameter circular BDD membrane, attached to a much thicker BDD mesh support frame (top surface polished using a resin bonded wheel), also 3 mm in diameter and containing 60 square shaped holes (100 μm × 100 μm), is shown in Figure 2a. Polycrystalline BDD was used as a support frame in order to keep the material properties associated with the frame and membrane as consistent as possible, although in principle any support frame material could be used. The optical image is obtained in reflection mode where the support frame is also visible. Figure S2, Supporting Information Section 2, shows the membrane in transmission mode, where the BDD mesh structure, akin to a standard TEM support, is evident. While most of the membrane lies flat on the support surface there are a few small raised areas, most likely arising from the transfer process, step (iv). Membrane transfer is the challenging step in the five stage fabrication process and is where future work will be directed. In total five BDD membranes were fabricated using this procedure, but there is the possibility to produce many more and increase the size of the membrane, if required. The number of samples which can be ion implanted during step (i) is limited only by the size of the ion implantation chamber.

**Figure 2:**
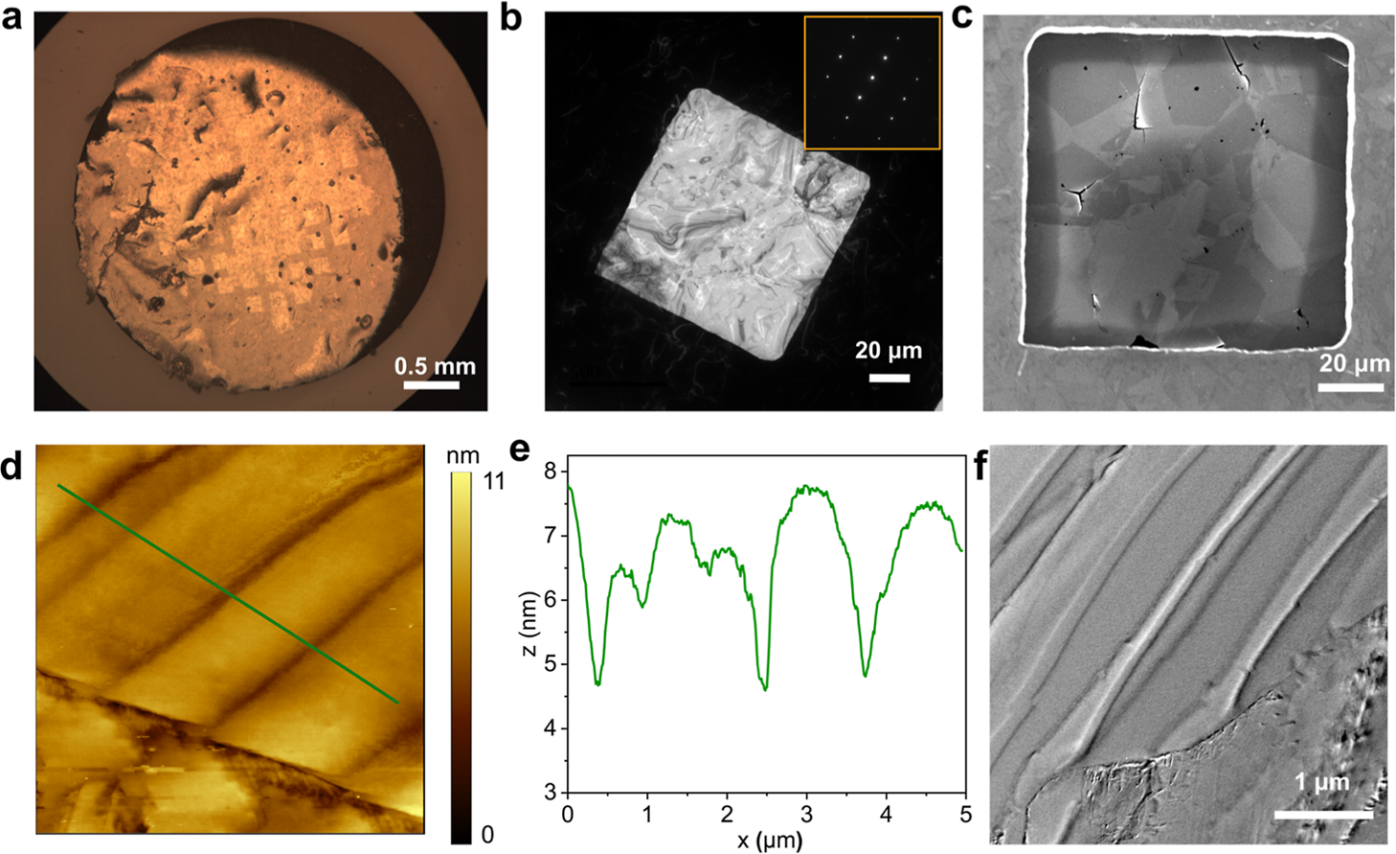
(a) Optical image of the lifted-off BDD membrane on the BDD mesh frame (imaged in reflection mode) prior to Ar/SF_6_ etching. (b) Low magnification bright field TEM image of one square region of electron beam transparent BDD, imaged at 200 kV. The inset to (b) is the electron diffraction pattern showing the (110) crystallographic orientation of the BDD membrane. (c) Corresponding SEM image to (b). (d) AFM topography image (5 μm × 5 μm) with (e) corresponding surface line profile, where *z* is the measured height and *x* is the position along the green line marked in (d). (f) TEM image, at 200 kV, demonstrating good electron transparency. The bright and dark diagonal lines are bend contours, while dislocations and a low-angle grain boundary are also present, lower right.

Figure 2b shows a low magnification bright field TEM image, at 200 kV, of the RIE etched BDD membrane in the area of one square-shaped mesh hole (100 × 100 μm) in the BDD frame. Clear contrast is seen between the bright area of the electron transparent membrane and the dark area of the thick frame. Shown in the inset to Figure 2b is the selected area electron diffraction (SAED) pattern for the etched membrane. A (110) crystallographic orientation is revealed which is independent of where the SAED pattern is recorded. The uniformity in crystallographic orientation is a result of both the polycrystalline BDD growth process which produces a dominant (110) texture, and the mechanical polishing procedure (prior to membrane fabrication) which reveals this texture.^19,27^ The SAED pattern recorded on the membrane highlights this dominant crystallography is retained during the membrane fabrication steps. This is a useful property of this material as it allows us to treat the BDD surface as a pseudo (110) single crystal for fundamental electrochemical studies.^40^

The grain structure, shown more clearly in the SEM image in Figure 2c, is also extremely useful for aiding identical location-TEM experiments or locating SECCM areas, post-measurement (vide infra). Figure 2c shows grains of different contrast separated by sharp boundaries. The darker grains indicate the more heavily boron doped grains, which are also the more recessed grains in the AFM images of Figures S3a and S4a,b, Supporting Information Section 3. Heterogeneous doping arises due to the different crystal facets taking up boron differently during the growth process. A schematic outlining the origin of the dominant ∼ (110) crystallography and the varying boron content across the membrane surface is shown in Figure S7, Supporting Information Section 4. Also evident in Figure 2c are a limited number of “defective” grain boundaries, likely containing non-diamond carbon. These have etched at a much faster rate compared to the surrounding BDD and appear as black lines in the SEM image. Figure 2d shows a higher resolution, 5 × 5 μm AFM image of the BDD membrane; the surface roughness is determined as 1.66 ± 1.24 nm (*n* = 3). Ripples are evident in the image, which occur due to internal stresses present in the thin diamond film, with cross sectional (height) information shown in Figure 2e. These are also evident in the TEM image in Figure 2f as bright and dark diagonal lines (bend contours), resulting from localized bending or curvature in the sample. TEM, SEM and AFM images for another square-shaped area of the membrane are shown in Supporting Information Section 4, Figures S8 and S9. Very similar features are observed.

In order to investigate the impact of Ar/SF_6_ dry etching on the polycrystalline BDD surface chemistry, XPS measurements were performed on two larger, 5 × 5 mm, and thicker (same material quality, polished using a resin-bonded wheel) BDD substrates, Supporting Information Section 5. One surface was the unetched control sample, while the other was Ar/SF_6_ etched. The relative surface compositions of C, O, F and S for the two BDD substrates are summarized in Table S4. C and O are present in the control surface, while C, F and S are present in the Ar/SF_6_ etched surface. The XPS C 1s and O 1s spectra for the BDD control (Figure S10a,b respectively) are as expected for the oxygen-terminated BDD used herein.^40^ The XPS C 1s, F 1s and S 2p spectra for the Ar/SF_6_ etched sample are shown in Figure S10c–e, respectively. The C 1s spectrum shows an intense central peak attributed to sp^3^ bonded diamond (78.5%), a small peak at lower binding energy due to sp^2^ bonded carbon (17.8%) and a higher binding energy sub peak associated with C–F_*x*_ (3.8%). In Figure S10d, the F 1s peak is visible at 687.21 eV,^41,42^ and is assigned to C–F bonding, corresponding to the C–F_*x*_ in the C 1s spectrum.^43,44^ In Figure S10e, the S 2p spectrum shows overlapping spin–orbit doublets with four peaks.^45^ The two dominant peaks at lower energy (163.68 and 164.86 eV) are most likely associated with the C–S–C bond,^46^ whereas the other two are attributed to the S–F_*x*_ bond (S–F, S–F_2_ or S–F_3_ are most likely). Details on the relative contributions and binding energies of the different compositions of the two surfaces are provided in Tables S5–S7 in Supporting Information Section 5.

While it is known that F-terminated diamond surfaces are hydrophobic^47^ it is interesting to consider what the addition of S does to surface hydrophobicity and the potential limits of the aqueous solvent window, Supporting Information Section 6. Contact angle measurements were conducted to assess the hydrophobicity/hydrophilicity of the freshly etched S/F terminated BDD surface. As shown in Figure S11a, this surface has only a slightly bigger contact angle of 69.7 ± 2.2° (*n* = 3) compared with hydrophilic, oxygen-terminated (control) BDD surface; 63.8 ± 0.1° (*n* = 3), demonstrating how S addition increases the BDD surface wettability (for water), compared to a F-terminated surface. Figure S11b, shows an electrochemical solvent window (first scan) recorded in 0.1 M KNO_3_ on the S/F terminated surface. Using a current density of 0.4 mA cm^–2^,^19^ the solvent window is ∼ 3.6 V, which is very similar to that for the control, oxygen-terminated BDD electrode of the same material quality (minimal sp^2^ carbon).^34^ This indicates that S/F surface termination does not significantly impact the electron transfer kinetics for water oxidation/reduction, when compared to oxygen termination. Future work will look to further explore the impact of S/F BDD termination (compared with the more traditional oxygen-termination) on the electrochemical response of a wide range of “inner sphere” redox couples.

### High Angle ADF STEM Analysis

To quantify the thickness of the thin membrane, electron energy loss spectroscopy (EELS) was employed.^19^ A 433 × 421 nm area of the membrane was selected and EELS spectra were obtained per image pixel (pixel size = 12 × 12 nm), corresponding to 1260 pixels in the area interrogated. The thickness, *t*, of the BDD membrane per pixel, can be determined using eq 1 ^48^

1 $$t=\lambda \ln\left(I_{t}/I_{0}\right)$$

where λ represents the total inelastic mean free path of electrons as a function of irradiation energy; 97.61 nm in diamond (for 200 kV irradiation).^49^ *I*_t_ is the total area under the whole spectrum and *I*_0_ is the area under the zero loss peak. A thickness histogram profile (Figure 3a) was extracted for each EELS spectrum recorded per image pixel, giving the mean thickness of the BDD TEM membrane as 49 ± 1 nm, *n* = 1260 pixels. EELS data recorded in another area of the membrane, shown in Figure S12, Supporting Information Section 7, for *n* = 870 pixels, gives a thickness value of 62 ± 6 nm. The data reveals a small variation in BDD membrane thickness, within the error of the EELS measurements. Going thinner is possible, achieved by increasing the etch time in step (v), appropriately.

**Figure 3:**
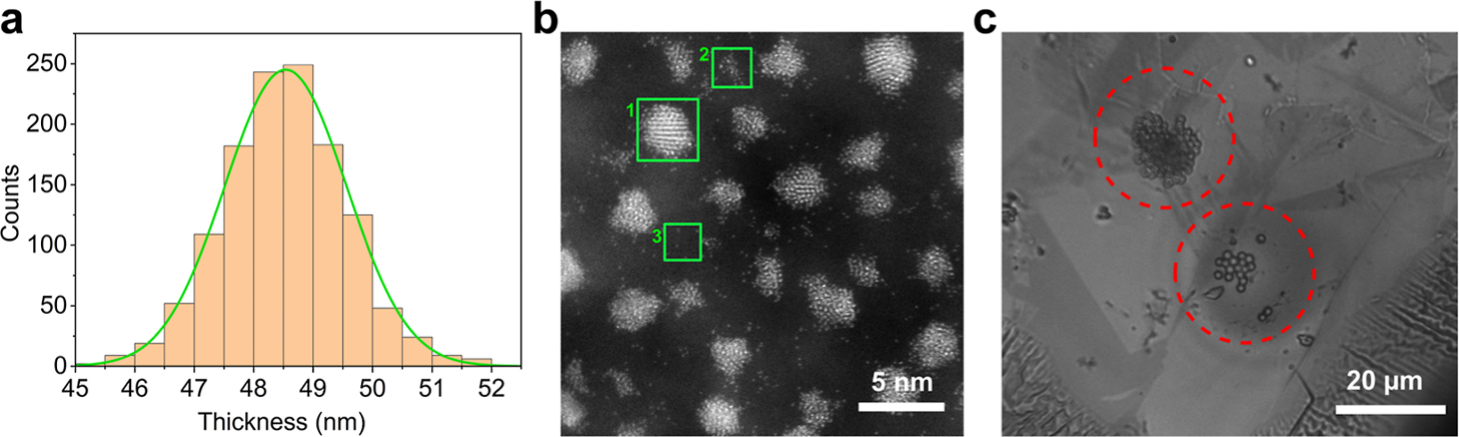
(a) The thickness histogram profile extracted pixel-by-pixel from EELS spectra of the BDD membrane. (b) ADF-STEM image of (1) crystalline gold NPs, (2) amorphous atom clusters, and (3) isolated single atoms, as identified in the green squares. (c) Transmitted white light optical image of polystyrene particles (∼1 μm in diameter) on the BDD TEM membrane.

To assess whether the BDD was thin enough to provide sufficient image contrast for imaging supported nanostructures, sputtering was used to produce gold nano-objects on the membrane surface. As the ADF-STEM image shows in Figure 3b, crystalline gold NPs, amorphous atom clusters and isolated single gold atoms are clearly evident (green squares) on the thin membrane.

### Optical Transparency

Heavily doped and thick BDD, as used herein as the starting material in the fabrication process, absorbs visible light so strongly it appears black with negligible optical transmission. However, by reducing the thickness of the material substantially, a significant fraction of the light can be transmitted. The optical absorption coefficient μ_λ_ (cm^–1^) at the wavelength, λ, determines how much the light is attenuated during transmission in accordance with eq 2, where *I*_λ_ is the transmitted light intensity, *I*_oλ_ is the incident light intensity and *t* is the thickness of the sample.

2 $$I_{\lambda }=I_{o\lambda }\exp\left(-\mu_{\lambda }t\right)$$

Room temperature optical absorption measurements (using a PerkinElmer Lambda 850 dual beam spectrophotometer) on single crystal BDD containing 22 ppm of boron (3.9 × 10^18^ boron atoms cm^–3^) gives a value for μ_λ_ (at 470 nm) of ∼ 17 cm^–1^. Assuming that the absorption coefficient scales with boron concentration, this results in an absorption coefficient of ∼ 1310 cm^–1^ for 3 × 10^20^ boron atoms cm^–3^ (appropriate to the material used herein). For a BDD membrane *t* = 50 nm, eq 2 predicts the attenuation of the light due to absorption is negligible (>99% of the incident light at 470 nm would be transmitted). However, in addition to absorption, light will be reflected at air-BDD interfaces. For normal incidence at such an interface, the reflection coefficient is

3 $$R=(n_{\lambda }-{1)}^{2}/(n_{\lambda }+{1)}^{2}$$

where *n*_λ_ is the refractive index at the wavelength, λ. At 470 nm, *n*_λ_ = 2.44,^50^ so *R* = 0.175 and when accounting for multiple reflections between each surface this leads to a reduced transmission of (1 – *R*)^2^/(1 – *R* ^2^) = 70.8%.

To experimentally understand the impact of losses on optical transmission through the BDD membrane, measurements were made with an FTIR microscope, as detailed in Supporting Information Section 8a. Over a longer wavelength range of 1.32–1.82 μm, (*n*_λ=1.5 μm_ = 2.39) a transmission of ∼ 54% was measured, only ∼ 17% less than the theoretical maximum, Supporting Information Section 8a. It is likely that absorption due to residual radiation damage defects from the ion implantation accounts for a fraction of these losses as the boron related optical absorption is still negligible in this wavelength range. Optical scattering caused by grain boundaries and surface roughness could also contribute.^51^ Optical transparent electrode (OTE) measurements made with BDD nanocrystalline films, 500–1000 nm thick, grown onto quartz, reported optical transmissions, in the visible region, of 50-60%.^52,53^ Slightly higher values were achieved using free-standing (thick) BDD which contained slot-shaped holes to transmit the light.^54^

To demonstrate optical transparency, microscopic visualization through the membrane was performed using an inverted light microscope. Fluorescent amine-modified polystyrene beads (∼1 μm in diameter), dispersed in water, were drop cast on the surface using a single-barreled SECCM pipet probe (∼40 μm in diameter), Supporting Information Section 8b. The sample was illuminated with white light in transmission mode, Figure 3c shows the resulting image of the surface. The ∼1 μm beads are clearly resolvable on the BDD surface (within the red dotted circles) along with the grain structure of the BDD membrane. Such measurements bode well for use of the thin BDD membranes for a wide range of OTE applications. BDD also has the advantage, that unlike the most common OTE, indium tin oxide, it does not suffer from degradation at cathodic potentials^55^ and has an extended cathodic potential window.^26^ Figure S14, Supporting Information Section 8b, shows the fluorescence image (illuminated using blue light, peak intensity wavelength 470 nm) of the fluorescent amine-modified polystyrene particles on the BDD thin membrane. The image was obtained in reflection mode but using an inverted microscope so light passes through the membrane. The beads are again clearly visible (white) against the black background, and importantly also demonstrates the low fluorescence background of the BDD membrane.

### SECCM Measurements of Gold Electrodeposition

SECCM allows localized electrochemical measurements in different positions across the BDD membrane. Here, we investigate how the applied electrode potential impacts the crystallinity and morphology of electrodeposited gold nanostructures using the SECCM–BDD–TEM platform. The SECCM setup for these experiments is described in Supporting Information Section 9. Cyclic voltammetry (CV) was initially used to investigate gold electrodeposition and dissolution in the SECCM configuration. Figure 4a shows representative first, second and third scan CVs in one spot, obtained on the BDD TEM membrane using an SECCM pipette tip of diameter ∼ 200 nm (measured using SEM, Figure S15, Supporting Information Section 9), at a scan rate of 0.5 V s^–1^. Scanning commenced at 0.80 V vs a Pd/Pd–H_2_ quasi-reference counter electrode (QRCE) and proceeded first in a negative direction. The pipette tip contained 20 μM of the gold salt HAuCl_4_, pH = 4.30, with no supporting electrolyte, to avoid salt crystallization in the meniscus footprints, that were subsequently analyzed by ADF-STEM.

**Figure 4:**
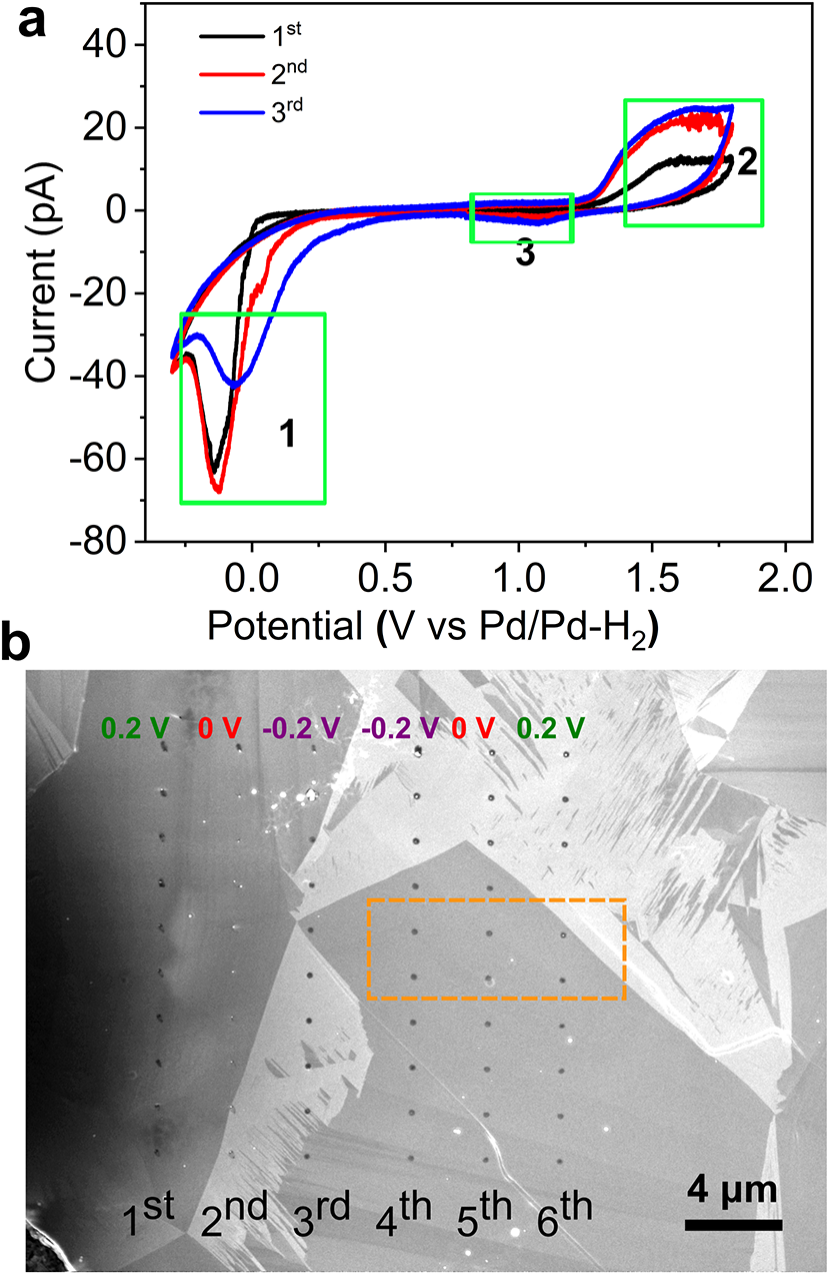
(a) Representative CVs (3 cycles) using the BDD–TEM–SECCM platform at a scan rate of 0.5 V s^–1^. The electrolyte was 20 μM HAuCl_4_ and the nanopipette diameter was ∼ 200 nm. (b) SEM images of the 60 SECCM meniscus footprints on the BDD TEM membrane, which are also used to estimate footprint diameter ∼ 500 nm. The grain structure of the BDD membrane is also evident. The six meniscus footprints in the orange rectangular area in (b) were analyzed by ADF-STEM.

The CVs exhibit shapes similar to those seen on other carbon electrodes for gold electronucleation and growth in acidic media.^56–58^ Gold electrodeposition peaks are labeled as 1 in Figure 4a. Upon scanning in the anodic direction, oxidation peaks (labeled 2) corresponding to gold oxidation and possibly gold dissolution (given chloride ions will be released into solution during AuCl_4_^–^ reduction^59^) are evident. Upon reversing the potential to scan again in the cathodic direction, small cathodic peaks (labeled 3) are observed, indicative of gold oxide reduction. A characteristic “nucleation crossover” is observed on the first scan, at −0.005 V, resulting from gold nucleation being easier on gold than BDD. Its absence on the second and third scans is most likely due to incomplete removal of gold from the BDD surface during the first scan. The onset potential for gold deposition is defined as the potential where ∼0.8 pA flows, which is just above that due to non-faradaic current processes of ∼0.75 pA. For the three consecutive scans, this potential shifts more positive with scan number (0.20 V for first, 0.51 V for second and 0.59 V for third scan), again as a consequence of gold electrodeposition on gold being easier than gold on BDD. It should be noted that the faradaic currents are larger than might be expected based on standard SECCM models,^60^ attributed to favorable meniscus wetting of the hydrophilic BDD substrate (vide infra), which leads to an increased electrode area and increased mass transport rates (e.g., evaporation-induced convection).

The effect of electrodeposition potential on the resulting gold nanostructures was further investigated by chronoamperometry for a fixed duration (0.5 s). Based on the CV measurements, three deposition potentials were chosen: +0.20 V; 0.00 V; and −0.20 V vs Pd/Pd–H_2_, all free from unwanted side processes such as hydrogen evolution. For each potential, a line of ten deposition spots was executed by moving the SECCM tip 3 μm between each electrodeposition. This process was repeated, resulting in six lines of SECCM electrodepositions and 20 measurements per deposition potential (Figure 4b). The position of the SECCM meniscus footprints can be seen using SEM, as shown in Figure 4b. The wetted diameter of the SECCM meniscus is typically ∼ 500 nm, which is larger than the size of the nanopipette diameter, due to the hydrophilicity of the BDD surface (Figure S11a, Supporting Information Section 6).^61^

To obtain information on the morphology and crystallinity of the gold nanostructures, analysis was focused on six meniscus footprints (orange rectangular box in Figure 4b) as the time scale is lengthy when using ADF-STEM to resolve all nanostructures within an individual footprint. All six footprints were chosen from a facet area with the same SEM contrast and indicative of a higher boron doped region. This was to rule out possible dopant level effects and includes two depositions at each of the three potentials, −0.20, 0.00, and 0.20 V vs Pd/Pd–H_2_. Figure 5 shows ADF-STEM images of the bottom three SECCM meniscus footprints (in the orange square, Figure 4b), at the three different deposition potentials. Lower magnification images are shown in Supporting Information Section 9, Figure S16, where the entire SECCM meniscus footprints were imaged with ADF-STEM. Particle size analysis using ImageJ was undertaken using the lower magnification images (Figure S16). The resulting average particle diameters are given in Table S8.

**Figure 5:**
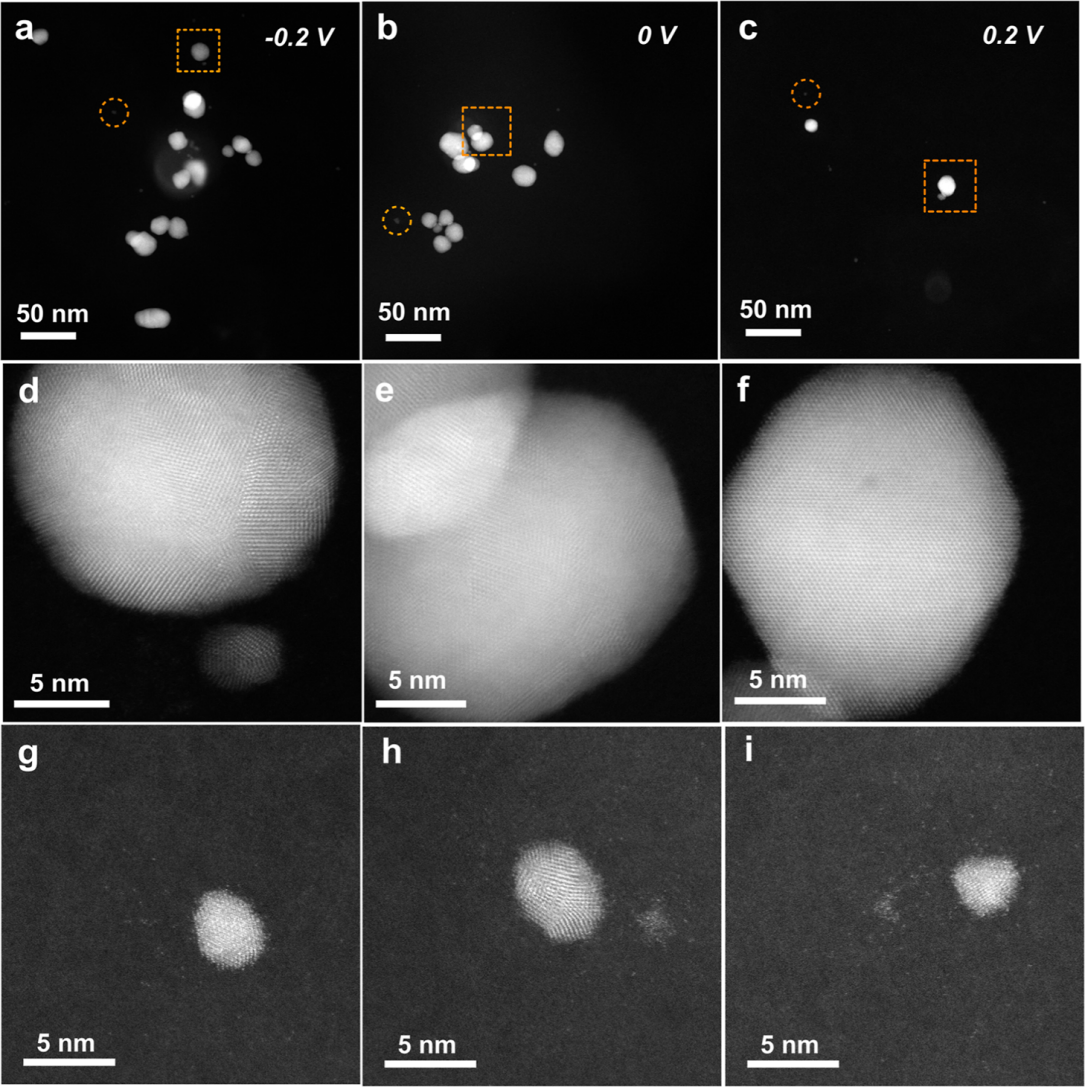
(a–c) ADF-STEM images of electrodeposited gold nanostructures on the BDD TEM membrane at SECCM chronoamperometry potentials of −0.20 V, 0.00 and 0.20 V vs Pd/Pd–H_2_ for 0.5 s. (d–f) Higher resolution ADF-STEM images of the gold NPs found in the zone denoted by the dotted orange squares in (a–c). (g–i) Higher resolution ADF-STEM images of smaller gold NPs, amorphous clusters and isolated single gold atoms, found in the zone denoted by the dotted orange circles in (a–c).

The smallest particles (13.8 ± 2.1 nm) are observed under the lowest (more positive) driving potentials, increasing in size (and polydispersity) from 18.2 ± 7.1 to 20.8 ± 10.9 nm, as the potential is made more negative (reducing). By increasing the magnification, nanostructures that were not immediately evident can now be seen. For example, the ADF-STEM images in Figure 5g–i, are recorded in the areas outlined by the orange dotted circles in Figure 5a–c. As Figure 5g–i show, smaller NPs, amorphous clusters with no crystalline order, and single isolated gold atoms are now visible. These smaller amorphous clusters and single atoms are likely to be unresolvable using alternative high resolution microscopy techniques such as AFM.^62^

A significant advantage of ADF-STEM (and TEM) over e.g. SEM and AFM is that information on NP crystallinity can also be obtained. ADF-STEM images of the gold NPs labeled by the orange dotted square boxes in Figure 5a–c are shown in Figure 5d–f. While crystal faceting (polycrystallinity) is revealed in the NPs shown in Figure 5d,e, at the lowest deposition potential (0.20 V), Figure 5f, the NP is a perfect single crystal (in the example shown, with (110) orientation). ADF-STEM was carried out on the electrodeposited NPs in the six meniscus footprints in order to investigate whether this trend holds when the crystallography of all NPs electrodeposited at the three different deposition potentials are examined. Table S9 summarizes the findings (for NPs of diameter ≥7 nm). For the highest driving force (−0.20 V vs Pd/Pd–H_2_), of the 45 electrodeposited NPs, none were found to be single crystal, and in some instances twinning was also observed. At 0.00 V, 6% (16 NPs investigated) were single crystal, with the number rising to ∼40% for the lowest driving force (8 NPs investigated). The number of electrodeposited NPs was also found to increase with increasing (negative) electrodeposition potential. This data indicates a correlation between driving potential and NP crystal quality, showing direct evidence for slower growth (lower deposition potentials) being preferred for defect free single crystals. Single-crystal metal NPs can exhibit superior electrical, mechanical and optical properties compared to their polycrystalline counterparts.^63–66^ The opposite is true if defective polycrystalline gold NPs are required, where increasing the driving force for electrodeposition is increasing the likelihood of non-ideal placement of atoms and the probability of growth defects such as twins forming.

### Comparison of BDD Membrane versus Carbon Thin Film for Correlative Microscopy Measurements

Finally it is useful to summarize the properties of the BDD membrane (supported on a BDD frame) compared against the common carbon film on Cu support, with the later primarily used in correlative SECCM and TEM measurements, as shown in Table 1. Complementary experimental details are given in Supporting Information Section 10. Table 1 includes assessment of (a) single gold atom resolution; (b) mechanical strength (elasticity) measured via indentation (deformation) AFM measurements, Supporting Information Section 10.1; (c) thermal stability in air (500 °C), Supporting Information Section 10.2; (d) high resolution TEM imaging at 200 kV at 400 °C (in situ), Supporting Information Section 10.3: (e) repetitive SECCM cycling into the sp^2^ carbon corrosion regime, Supporting Information Section 10.4; (f) ease of use for correlative SECCM–TEM. As shown in Table 1, the BDD membrane exhibits a superior performance over the carbon film in all areas, except resolution, where both are similar.

**Table 1:** Comparison of the Performance of the BDD Membrane Evaluated Against the Carbon Film

measurement	BDD membrane (∼50 nm)/BDD support	Carbon (C) film (∼20–30 nm)/ Cu support
single gold atom resolution (ADF-STEM)	yes	yes
mechanical strength (AFM tests)	stronger than C film	weaker than BDD membrane
thermal stability in air (500 °C)	stable—heating also helps clean the surface	unstable—C film disappears, Cu undergoes morphological transformation
high resolution TEM imaging at 200 kV at 400 °C (in situ)	no obvious change	C film thins and thinned region undergoes graphitization
repetitive SECCM cycling into the sp^2^ carbon corrosion regime	no obvious change	evidence of C corrosion, film weakens, cracks in the film emerge
ease of use for correlative SECCM–TEM	very stable surface, can be imaged many times (S/TEM) without change to surface or gold deposit; false engagements do not result in puncturing of the membrane; inherent grain structure makes identical location experiments easy; no damage to the membrane when handling with tweezers	surface can contaminate more easily, tip can break through film with a false engagement; surface uniformity makes identical location more challenging; tweezer contact with the C film results in damage

## Conclusion

Fabrication of thin (∼50 nm), free-standing, polycrystalline BDD membranes (>5 mm^2^ in area), using an ion implantation and anneal, lift-off and RIE etch procedure was reported. The membranes were produced from high quality (minimal sp^2^ carbon content) polycrystalline (grain size ∼ tens of μm) BDD which displayed a wide (and low background current) solvent window in aqueous solution. AFM measurements revealed a surface roughness of ∼ 1.7 nm RMS. The membrane was shown to be electron and optically transparent (>50% transmission), with the latter providing opportunities for correlative spectroelectrochemical measurements, over a wide potential range, free from corrosion and chemical degradation. The superior properties of the membrane over the more commonly used carbon film, for correlative SECCM (or electrochemical)–TEM measurements, were also demonstrated.

Our developments also open up possibilities for BDD membrane integration in e.g. operando X-ray absorption spectroscopy^67^ or as an alternative to graphene in in situ electrochemical TEM,^68^ however further work would be required. The nanofabrication procedure is equally applicable to the production of single crystal BDD membranes for correlative microscopy and scalable such that the production of larger area membranes is also possible. Increasing etch times will also facilitate the production of thinner BDD membranes which will expand the range of lower atomic number elements which can be imaged at the level of single isolated atoms.

To explore the impact of electrode potential on the number density, size and crystal quality of gold NPs formed during electrodeposition, correlative microscopy experiments were carried out using SECCM and ADF-STEM. Systematically reducing the electrodeposition driving potential was found to increase the propensity toward defect free single crystal NPs, without the need for targeted molecular precursors.^66^ For all electrodepositions, isolated single atoms and atom clusters were also seen when imaging at higher resolution. While experiments were focused on NP deposition in areas of high dopant density on the BDD membrane, given the heterogeneous doping of the membrane, future work will explore the impact of the boron doping level on the electrochemical process of interest. Such platforms offer tremendous opportunities for the correlation of nanostructure with (electro)­activity, and understanding how electrochemical deposition parameters can be tuned to engineer specific structures at the atomic level.

## Experimental Section

### Materials

All solutions were prepared from ultrapure water (>18.2 MΩ cm, Milli-Q, Millipore Corp.). The lift-off etch solution comprised 50 mM sulfuric acid (H_2_SO_4_, >96%, Merck, UK). For SECCM electrochemical measurements, 20 μM HAuCl_4_ (Sigma-Aldrich) was used. A free-standing polycrystalline BDD plate, ∼ 500 μm thick, was employed as the starting material for all experiments. The BDD was grown using microwave chemical vapor deposition (Element Six, Harwell, Oxford, UK) suitably metallically doped with boron ∼ 3 × 10^20^ B atoms cm^–3^ and containing minimal sp^2^ carbon content; electrode E in ref 34. The BDD was removed from the nondiamond growth substrate and the growth face polished on a resin-bonded wheel (AFM data in Figures S3a and S4a,b). The plate was cut into 3 mm diameter disks using laser micromachining (355 nm Nd:YAG 34 ns laser, Oxford Lasers E-Series), to mimic the geometry of commercial carbon TEM grids. The samples were acid cleaned to remove machining debris by immersing in concentrated H_2_SO_4_ (>96%, Merck, UK) saturated with KNO_3_ (99.97%, Sigma-Aldrich, UK) at ∼ 200 °C for 30 min, followed by another 30 min in concentrated H_2_SO_4_ at ∼ 200 °C before rinsing with ultrapure water.^69^

### Fabrication of Thin BDD Membranes

The polished growth face of the BDD was ion implanted as described in Supporting Information Section 1. The implanted region was converted to sp^2^ carbon by annealing at 1300 °C for 2 h,^70^ followed by acid cleaning using the same protocol described above. The sp^2^ carbon layer was etched away to “lift-off” the BDD membrane using electrochemical etching,^36^ as detailed in Supporting Information Section 11, along with an accompanying video of the process. The lifted-off BDD membrane was attached to a BDD circular mesh frame as described in Supporting Information Section 2. The same material (BDD) was chosen as the membrane carrier, due to its conductive nature and mechanical robustness. Bespoke laser micromachining of the carrier also meant the mesh size could be tailored to the application of interest. Finally, an optimized dry etching process was employed, described in Supporting Information Section 3, to thin the membrane down to less than 100 nm in order to achieve electron (and optical) transparency. Typically, ICP-RIE etch times of 11 min were employed (*n* = 5).

### TEM Characterization

TEM and electron diffraction of the BDD TEM membranes were carried out using a JEOL JEM 2100 TEM at 200 kV. STEM images were recorded using a double aberration-corrected JEOL JEM-ARM 200F TEM operated at 200 kV. To estimate the thickness of the BDD TEM membrane, EELS spectra were collected in STEM mode, at a probe convergence semiangle of 32 mrad, a spectrometer semicollection angle of 25 mrad, and a dispersion of 0.25 eV per channel. To obtain accurate threshold energies for ionization edges, the dual EELS mode was used. The energy resolution according to the full width at half-maximum measured at the zero-loss peaks was ∼ 1.8 eV at an electron probe current of ∼ 200 pA.

### Surface Characterization

#### AFM

Surface roughness measurements were made using an AFM (Innova, Bruker, USA) operating in ScanAsyst mode with image analysis performed using Gwyddion 2.5.^71^ For each sample, at least three areas were characterized in order to provide representative data.

#### SEM

Field emission SEM was used to image the BDD TEM membranes. Images were recorded using the in-lens, secondary electron, and STEM detectors on a Zeiss Gemini FE-SEM 500 operating at 18 kV.

#### XPS

XPS was conducted using an Omicron Nanotechnology Multiprobe instrument spectrometer with a monochromated Al Kα X-ray source (1486.69 eV) and a SPHERA hemispherical electron energy analyzer, in a chamber with a base pressure below 1 × 10^–10^ mbar. More specific experimental details are found in Supporting Information Section 5.

#### Contact Angle Measurements

Contact angle measurements were recorded using a drop shape analyzer (DSA100, Krüss Scientific, Germany) with an ultrapure water droplet of volume 8 μL. Measurements were recorded in triplicate, with the surface dried carefully between each recording using a lint free tissue, Supporting Information Section 6.

### Gold Sputter Deposition

For initial tests of STEM resolution of metal nanostructures, gold was sputter deposited onto the BDD TEM membrane using a NanoPVD system, Moorfield, UK with 15% direct current power supply unit (DC PSU), pressure 8 × 10^–3^ mbar for 1 s.

### SECCM Setup and Electrochemical Measurements

All SECCM experiments were carried out on a home-built scanning electrochemical probe microscopy workstation as detailed in Supporting Information Section 9.1.

## Supplementary Material





## Notes

Ion implantation simulations; transfer BDD membrane on the BDD mesh frame; dry etching; BDD growth morphology and additional characterization data of BDD membrane; XPS data; contact angle and electrochemical solvent window measurements; EELS analysis; optical measurements; SECCM setup and electrochemical measurements; details on the different measurements made for assessment of the BDD membrane versus C thin film; electrochemical etching setup for membrane lift-off (PDF)
An accompanying video of the membrane electrochemical etch process for lift off (MP4)
