# Supplementary material for: Optical and Electron Transparent Polycrystalline Boron Doped Diamond Membranes for Nanoscale Correlative Structure-Electrochemical Measurements
Source: ACS Nano. 2025 Dec 3;19(49):41637–47. doi: 10.1021/acsnano.5c13873 (PMC12713776; doi:10.1021/acsnano.5c13873)
Supplement: Supplementary file 1 [file nn5c13873_si_001.pdf]

Supporting Information

Optical and Electron Transparent Polycrystalline Boron Doped Diamond Membranes for Nanoscale Correlative Structure-Electrochemical Measurements

Pei Zhao,[a] Daniel Houghton,[a] Joshua J. Tully,[a] Dimitrios Valavanis,[a] Patrick R. Unwin,[a] Richard Beanland,[b] Yisong Han,[b] Marc Walker,[b] Mark E. Newton,[b] Julie V. Macpherson[a,]*

[a]Department of Chemistry, University of Warwick, Coventry, CV4 7AL, UK

[b]Department of Physics, University of Warwick, Coventry, CV4 7AL, UK

SI 1. Ion implantation simulations

SI 2. Transfer BDD membrane on the BDD mesh frame

SI 3. Dry etching

SI 4. BDD growth morphology and additional characterization data for the BDD membrane

SI 5. XPS data

SI 6. Contact angle and electrochemical solvent window measurements

SI 7. EELS analysis

SI 8. Optical measurements

SI 9. SECCM setup and electrochemical measurements

SI 10. Details on the different measurements made for assessment of the BDD membrane versus C thin film

SI 11. Electrochemical etching set-up for membrane lift-off

SI 1. Ion implantation and simulations

The BDD was ion implanted (Ion Beam Centre, University of Surrey, UK) with a dose of 2 × 10^{16} carbon ions at 2 MeV cm^{-2}. A stopping and range of ions in matter (SRIM) simulation was used to estimate the approximate depth and width of the end-of-range damage layer for ion implantation.[1, 2] In diamond, there is a critical threshold of damage, D_c, described as vacancies per cm^3, where the diamond will convert to sp^2 bonded carbon under high temperature annealing (1300°C). For single crystal diamond, D_c values in the literatures range from 1 × 10^{22} to 9 × 10^{22} vacancies per cm^3;[3-5] no values are provided in the literature for polycrystalline BDD. SRIM simulations usually overestimate the true vacancy density as they do not take into account dynamic annealing[6] or damage saturation effects[7] in the material. Therefore, any results obtained using SRIM are at best an estimate. The resulting SRIM simulation is shown in Figure S1. The data, assuming a D_c value of 2.8 × 10^{22} vacancies per cm^3,[8] indicates a damage region which begins ~ 0.9 μm below the surface and is estimated to be ~ 0.4 μm thick.

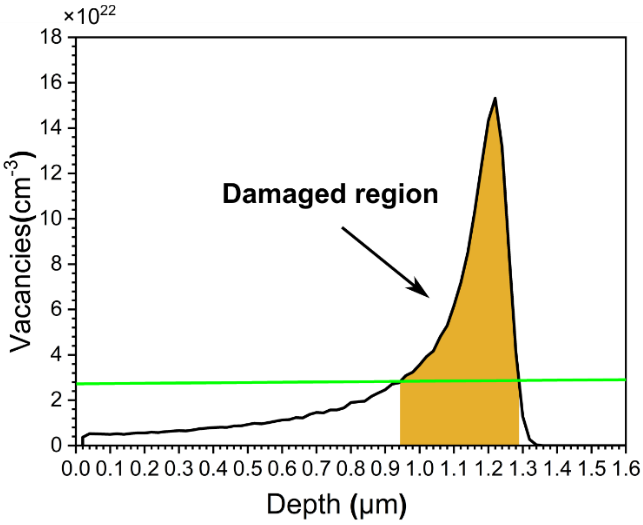

Figure S1. Vacancy density plot for the implantation of 2 MeV carbon ions at a dose of 2 × 10^{16} cm^{-2} into single crystal diamond. The green line indicates the minimum vacancy concentration threshold for graphitisation. The orange shaded area indicates the region that is damaged.

SI 2. Transfer of BDD membrane to the BDD mesh frame

A 3 mm diameter disk of BDD (~70 μm thick) was laser micromachined into a frame pattern containing 100 μm × 100 μm squares, with a square to square spacing (measured from the central point of each square) of 180 μm. The top and bottom surfaces were resin-bond polished to low surface roughness, ~ 1 nm RMS. The frame was acid cleaned (as described in the main text) to remove machining debris. The home-made frame was laser scribed with three squares at the periphery of the frame to aid identification of the SECCM droplets to the resulting TEM image.

As the BDD membrane is thin ca. 900 nm, care must be taken when transferring to the frame. After lift-off, the membrane is very loosely attached (physiosorbed) to the underlying BDD substrate. First, a small amount of crystal bond glue was dissolved in acetone (~2 mL). Using a syringe, two droplets of the solution were placed on the BDD frame, which sits on a glass slide. The "stamp" which is the BDD membrane and underlying substrate, was inverted and pressed gently down onto the frame. After a few seconds, the "stamp" was lifted slowly, the detached membrane remains fully adhered to the BDD frame. Finally, the frame and membrane were rinsed by dipping in acetone to remove residual crystal bond and left to dry in a desiccator under vacuum, ready for dry etching. An optical image of a lifted-off BDD 3 mm diameter membrane, attached to a BDD support is shown in Figure S2.

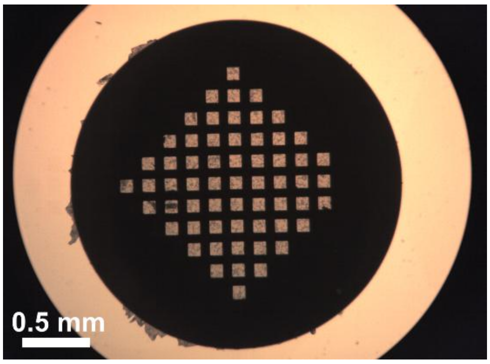

Figure S2. Optical image of the BDD membrane on the BDD mesh frame with sixty, 100 μm × 100 μm squares (imaged in transmission mode).

SI 3. Dry etching

3.1 Etching tests

Inductively coupled plasma reactive ion etching (ICP-RIE) was employed to uniformly thin the BDD membrane whilst at the same time maintaining low surface roughness.[9, 10] As studies to date have focused on etching of single crystal diamond surfaces, a preliminary investigation using this polycrystalline BDD material (but in much thicker form due to ease of handling) was first undertaken using different etch gases.

Three different etch recipes commonly used with single crystal diamond were investigated; (i) O_2, (ii) Ar/Cl_2 and (iii) Ar/SF_6.[9] Initial studies investigated etch rates and surface roughness. Prior to etching, the resin bonded polished samples were rinsed for 10 mins in ultra-pure water, cleaned in acetone in an ultrasonic bath for 3 mins and blown-dry with nitrogen. This procedure was repeated but replacing acetone with isopropyl alcohol (IPA). Finally all substrates were ashed using a soft O_2 ash recipe (radio frequency = 50 W, 50 m Torr, 5°C, 100 sccm O_2, 2 min). If the surface is not clean, micromasking can occur during etching which impacts surface finish.[9, 11]

After cleaning, half of the sample was masked using Kapton tape, and the substrate attached to a fused silica wafer. Different etches were carried out using ICP-RIE (Corial 200IL). To keep the temperature in the reaction chamber low, a 5 min cool down step under 100 sccm Ar at the same pressure as the corresponding recipe was used after each 5 min of etching. A summary of the etching recipes employed is found in Table S1.

Table S1. Summary of ICP-RIE etch parameters.

**Dry Etching Recipe**	Ar/Cl_2 [9]	Ar/SF_6 [12]	O_2
**ICP / RF power (W)**	500 / 200	700 / 100	700 / 100
**Pressure (m Torr)**	7.5	9	10
**Gases Flow (sccm)**	10 Ar / 20 Cl_2	20 Ar / 10 SF_6	30 O_2
**Temperature (°C)**	5	5	5

Duration of Etch (min)*	20	15	3

**5 minutes cooling under 100 sccm Ar after every 5 minutes etch.*

A white light interferometer (Profilm3D, Filmetrics, USA) was used to measure the step height between the unetched (surface under the Kapton tape) and etched area. The image was analyzed using ProfilmOnline. Two substrates for each recipe were initially used to compare and estimate etch rates, as summarized in Table S2. The etch times were based on reported etch rates for single crystal diamond.[9, 11, 12] The data demonstrates that O_2 has the highest rate (255 nm / min based on n = 2), similar to that reported previously for single crystal diamond. The etch rates for Ar/Cl_2 or Ar/SF_6 are slower; 59 nm / min and 86 nm / min respectively (n = 2).

Table S2. Summary of etch results.

Dry Etching	Sample	Etch time (min)	Step height (µm)	Etch rate (nm / min)	Average etch rate (nm / min)
**Ar/Cl₂**	#1	20	1.30	65	59
	#2	20	1.05	53	
**Ar/SF₆**	#1	15	1.20	80	86
	#2	15	1.37	91	
**O₂**	#1	3	0.72	240	255
	#2	3	0.81	270	

3.2 Surface topography

To investigate the impact of the three etch gases on the polycrystalline BDD surface topography, AFM was used to characterize surface topography before and after etching. All RMS roughness values are quoted as mean ± SD and calculated from n = 3 areas per sample, unless otherwise specified.

Figure S3a shows a typical 50 ×50 μm image of the resin-bonded polished polycrystalline BDD surface prior to etching. For the surface before etching, different grain structures are clearly evident. The very small height variations are due primarily to boron dopant density. The higher boron doped areas (darker regions) are mechanically softer and therefore polish faster, leading to recessed surfaces compared, to the lower boron doped grains.[13] On an individual grain e.g. for the 5 × 5 μm area (green square) in Figure S3a, the surface roughness is 1.50 ± 0.04 nm; *n* = 3. Over 50 ×50 μm, this value increases to 9.08 ± 0.52 nm due to higher doped regions of the heterogeneously doped surface polishing at a faster rate and producing steps between high and slightly less doped regions.

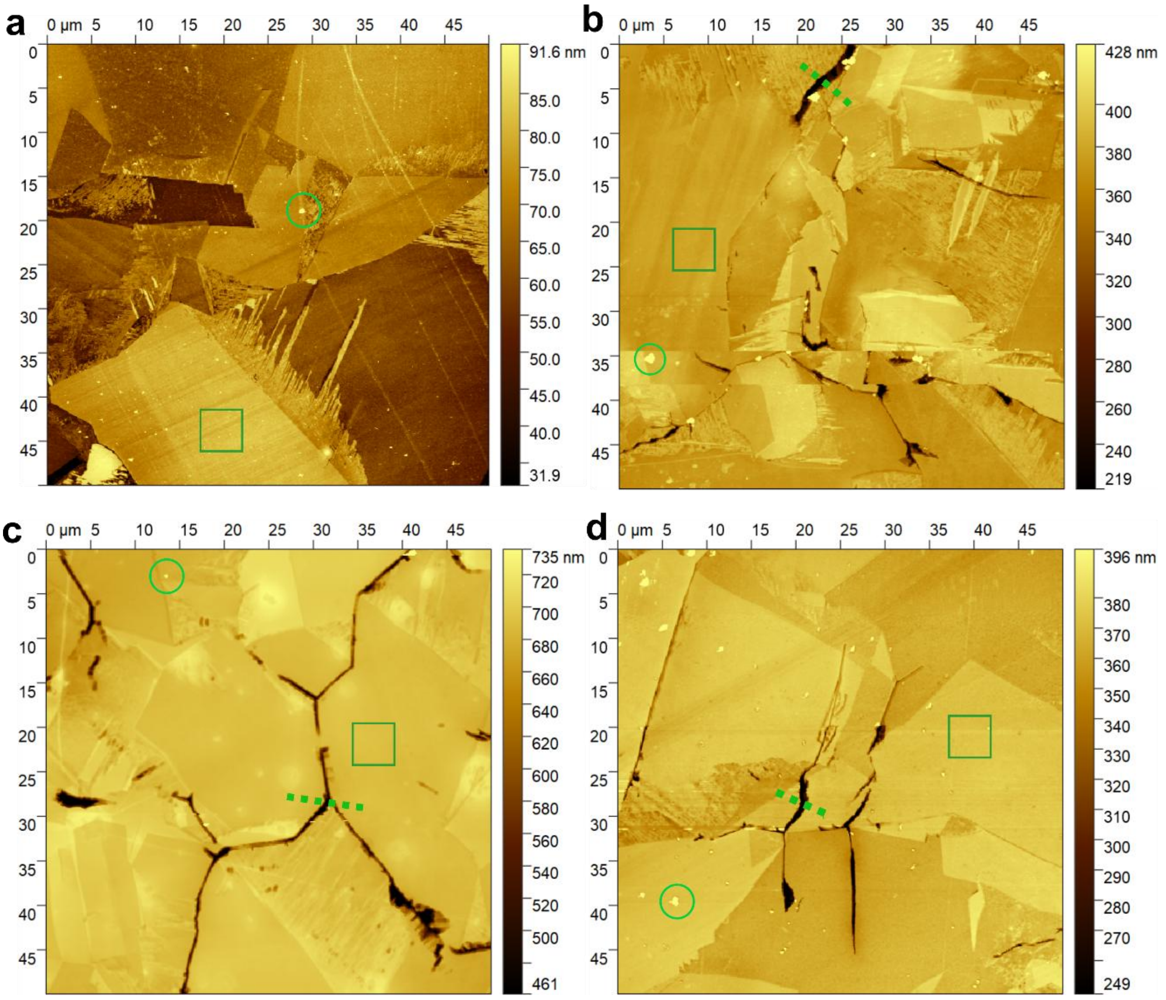

Figure S3. Representative AFM images of a polycrystalline BDD surface (50 μm × 50 μm) (a) before etching. (b) After O_2 etching. (c) After Ar/Cl_2 etching. (d) After Ar/SF_6 etching. Note the green squares represent 5 μm × 5 μm areas for one grain only, the green dotted lines indicate trenches and the green circled areas, possible contamination.

Figures S3b, c and d shows the topography of the BDD surface (50 ×50 μm) after the ICP-RIE etch using O_2 (3 min etch), Ar/Cl_2 (20 min etch) and Ar/SF_6 (15 min), respectively. After O_2 etching the surface roughness on a grain has slightly increased; 1.93 ± 0.30 nm (n = 3), whilst that for the 50 ×50 μm area has increased more significantly to 16.83 ± 5.50 nm (n = 3), most likely due to trench formation at the most defective grain boundaries. This occurs due to preferential etching of this material, and is present for all etches (Figures S3 and S4). For Ar/Cl_2, the 50 ×50 μm surface roughness is greater, 24.21 ± 8.25 nm (n = 3), due to the more significant impact of trench formation. However, interestingly the on-grain roughness is lower: 1.16 ± 0.34 nm (n = 3) Finally, for Ar/SF_6, whilst trenches at grain boundaries are present, the number appears reduced, leading to the smoothest 50 ×50 μm surface roughness of 11.92 ± 2.06 nm (n = 3). The surface roughness of individual grains is also very similar to the pre-etched surface; 1.52 ± 0.65 nm (n = 3). Table S3 provides a summary of RMS surface roughness as a function of etch gas for an individual grain (5 × 5 μm) and sampling the entire area (50 × 50 μm).

Table S3. Summary of RMS surface roughness values over the entire area (50 × 50 μm) or on an individual grain (5 × 5 μm).

BDD	Area size 50 × 50 μm / 5 × 5 μm	RMS of area 1 (nm)	RMS of area 2 (nm)	RMS of area 3 (nm)	RMS surface roughness (nm)*
**Before etching**	50 μm	9.24	9.51	8.50	9.08 ± 0.52
	5 μm	1.50	1.54	1.46	1.50 ± 0.04
**After Ar/Cl_2 etching**	50 μm	31.97	15.55	25.10	24.21 ± 8.25
	5 μm	1.47	0.79	1.23	1.16 ± 0.34
**After O_2 etching**	50 μm	22.57	16.32	11.60	16.83 ± 5.50
	5 μm	2.22	1.94	1.62	1.93 ± 0.30
**After Ar/SF_6 etching**	50 μm	14.06	11.76	9.94	11.92 ± 2.06
	5 μm	2.26	1.22	1.07	1.52 ± 0.65

Two other areas were imaged to complement Figure S3, and are shown in Figure S4. Small particles marked with the green circles are likely to be contamination particles, possibly introduced during handling/cleaning of the samples. They are also evident in literature published AFM images of single crystal diamond.[14]

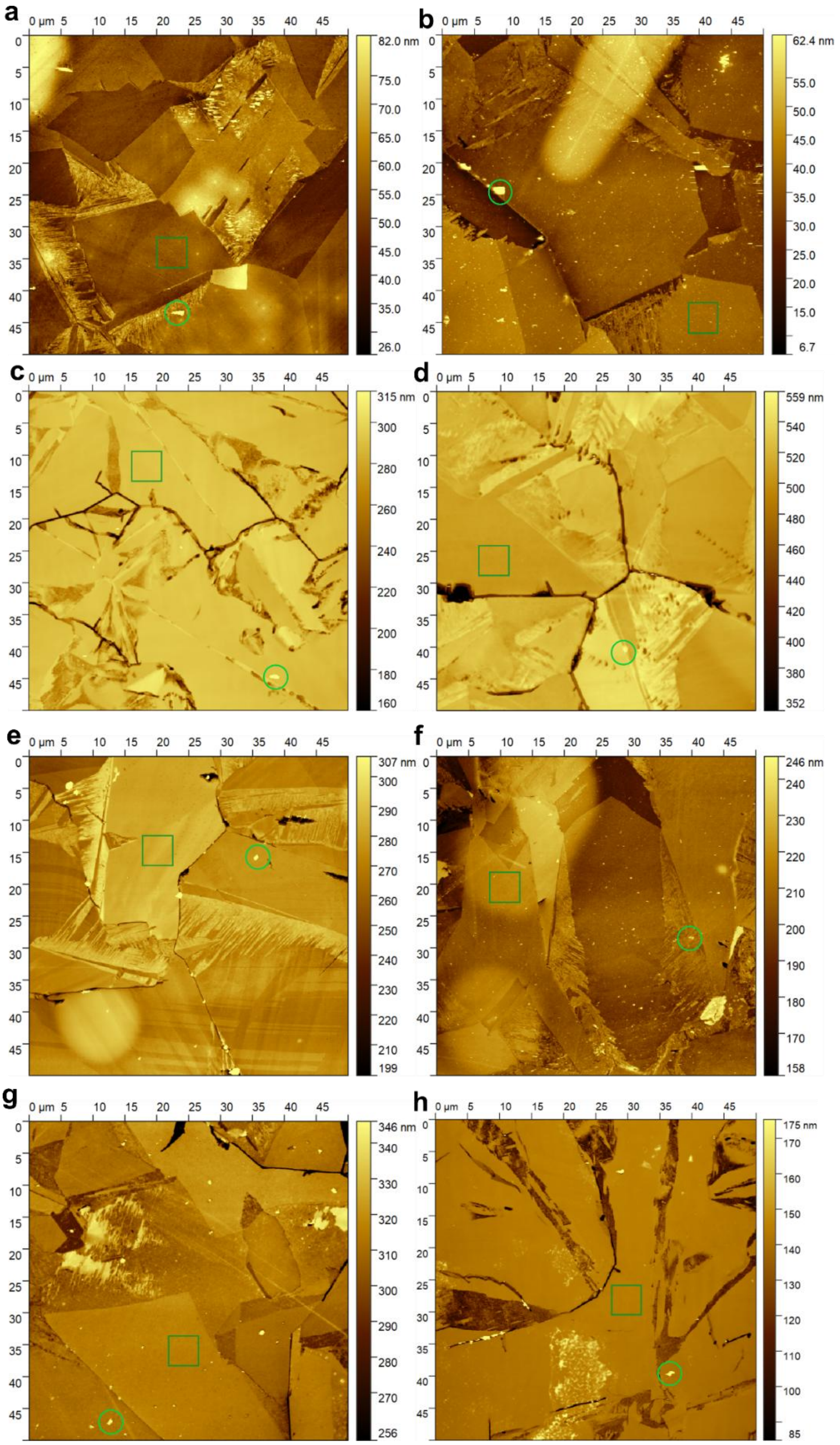

Figure S4. AFM images of BDD surfaces (50 µm by 50 µm) (a, b) before etching. (c, d) after Ar/Cl_2 etching, (e, f) after O_2 etching, (g, h) after Ar/SF_6 etching. Note that the green squares represent an on grain region of 5 µm by 5 µm and the green circles represent areas of possible contamination.

After etching, the very dark regions of the AFM image represent grain boundaries that have etched significantly to form "trenches". This is likely due to the fact that depending on the nature of the grain boundary, defective diamond and sp^2 carbon may be present.[15] The AFM data in Figures S3b-d, and Figure S4c-h, are also useful in identifying the most defective grain boundaries, as these are the sites which etch most rapidly.

AFM cross sectional measurements of etched trenches on the BDD surface (green dotted lines in Figures S3b, c and d) are shown in Figure S5 for the three differently etched surfaces. Penetration depths will be impacted by the AFM tip geometry.

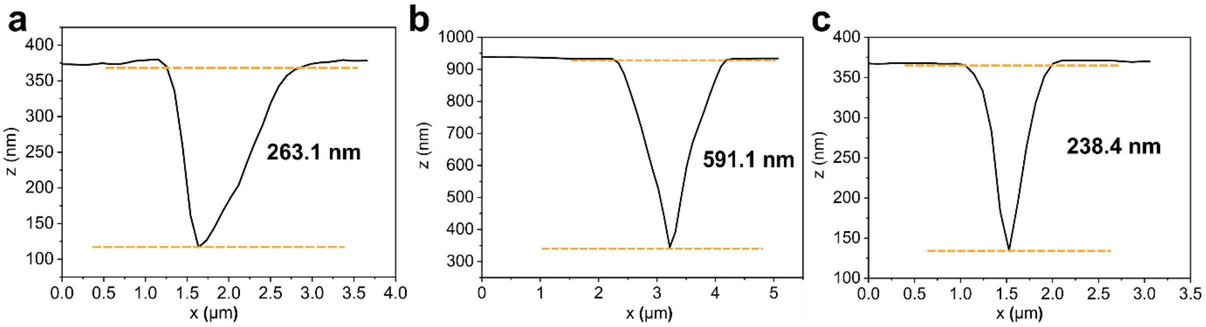

Figure S5. Cross-sectional height profiles corresponding to the green dotted lines (trenches) in Figure S3b, c and d, respectively.

3.3 Etch rate under Ar/SF_6

Based on the results, Ar/SF_6 was chosen as the RIE etchant given its moderate etch rate which offers better control when thinning to electron beam transparency and low surface roughness. To better quantify the etch rate data and gain more accurate control, experiments were conducted on ten polycrystalline, polished (using a resin-bonded wheel) BDD substrates. The substrates were half-masked by Kapton tape and etched using the Ar/SF_6 etch recipe for times in the range 2 to 20 min, increasing by 2 min for each sample. The resulting step height across the etched and unetched surface was measured using white light interferometry. The results of etch time versus etch depth are plotted in Figure S6, which indicates a linear relationship (R^2 = 0.989) with an etch rate of 87 ± 3 nm / min.

For membrane etching, based on this data an initial Ar/SF_6 etch time of 9 mins was employed and the membrane placed in the TEM to assess electron transparency. If no electron beam transparency was evident (indicated qualitatively by the brightness of the image) the etch time was increased by a further 30 s and the membrane reassessed using TEM. This continued until electron transparency was achieved.

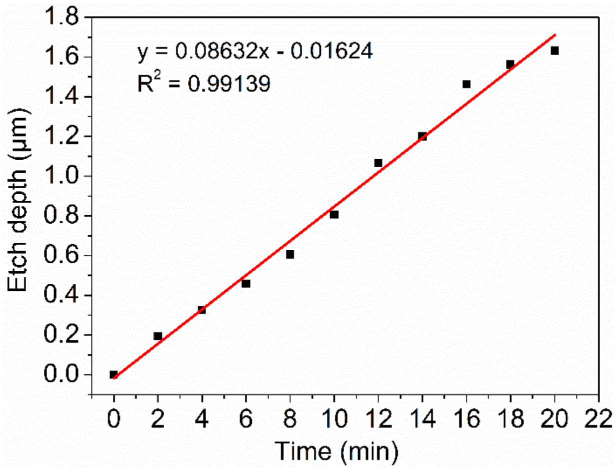

Figure S6. Plot showing etch time versus etch depth for an Ar/SF_6 etch of BDD.

SI 4. BDD growth morphology and additional characterization data for the BDD membrane

During polycrystalline BDD growth, the surface grows with a dominant (110) texture. In Figure S7a the (110) direction is pointing upwards as we look down onto the (111) and (100) crystal faces of the polyhedron. During growth, different crystal faces take up boron differently with (111) > (110) > (100).[16] If the top surface is now subject to a polish using a resin bonded wheel, the (110) face is revealed, as shown in the perspective view of Figure S7b. However the exposed face would contain different levels of boron doping reflective of the original growth morphology and crystal facets. Figure S7c represents a smaller rectangular area of the polished region, which has a dominant (110) crystal orientation, but contains different boron doping levels due to the original (100) and (111) crystal facets, with dark blue indicating high levels and light blue lower levels.

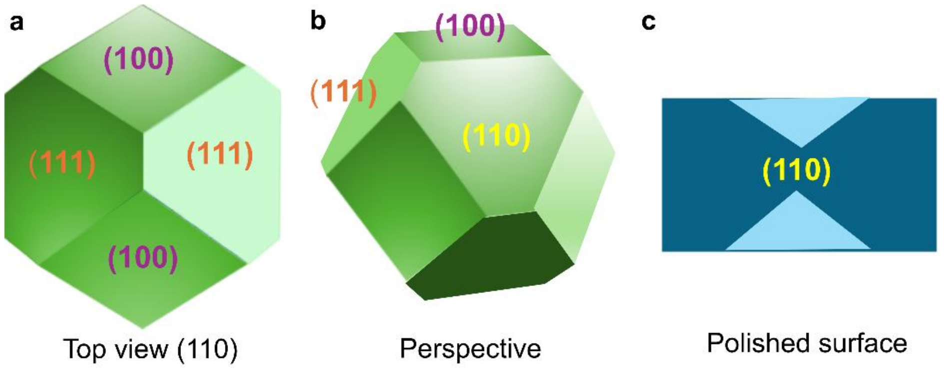

Figure S7. (a) Initial BDD growth polyhedron after (b) polishing on the top surface to reveal the (110) face.[17] (c) Illustrative example of the variation in boron doping level on this polished (110) surface, reflective of the original doping levels of the (100) and (111) facets.

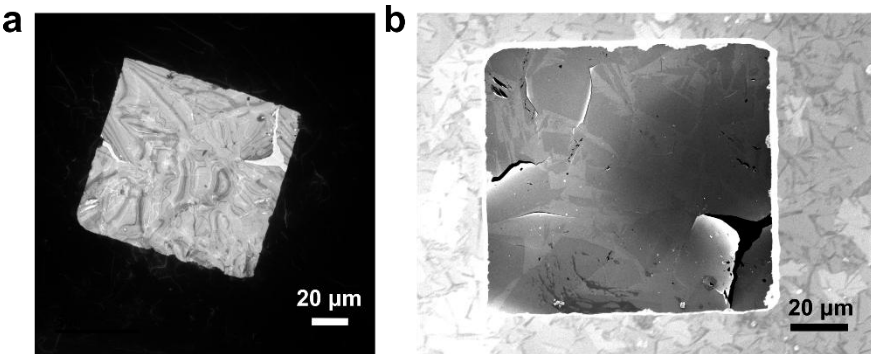

Figure S8. Electron transparent BDD membrane on the frame (a) low magnification TEM image of a square area with (b) the corresponding SEM image.

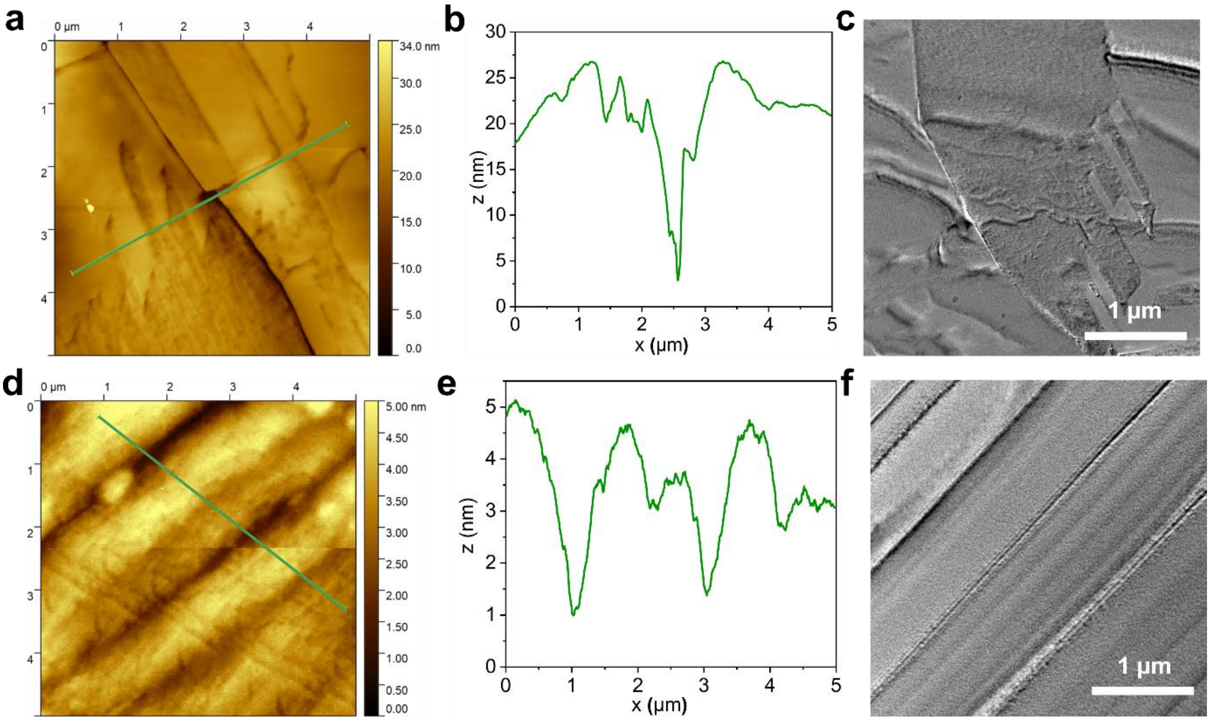

Figure S9. (a, d) AFM topography images (5 µm × 5 µm) of two different areas of the electron beam transparent BDD membrane, with (d, e) corresponding surface line profile, where *z* is the measured height and *x* is the position along the green line marked in (a, d). (c, f) TEM images, where bend contours are evident.

SI 5. XPS data

XPS measurements were performed to explore the chemical composition of the control oxygen terminated BDD surface and Ar/SF_6 etched BDD surface. Samples were mounted to Omicron-style flag plates using strips of Ta foil across the corners of the sample. XPS measurements were made at a take-off angle of 30° with respect to the surface plane; this corresponds to a penetration depth of 4.9 nm (calculated using the QUASES-IMFP-TPP2M software v3.0[18]).[19, 20] All XPS measurements were taken after annealing the samples at 500°C under ultra-high vacuum conditions in order to remove any surface adsorbed contaminants.[14] This annealing temperature is not high enough to decompose surface oxides.[14, 21] Survey spectra were acquired using a pass energy of 50 eV while high-resolution core level spectra were acquired at a pass energy of 10 eV (resolution approximately 0.47 eV). All measurements were performed using an analysis area 1.1 mm in diameter. All data collected were fitted in CasaXPS using Voigt (mixed Gaussian-Lorentzian) lineshapes and Shirley backgrounds, with asymmetry included for the sp^2 bonded carbon peak which was fitted using an asymmetric Gaussian-Lorentzian line-shape. Prior to measurements, the spectrometer transmission function and work function were calibrated using sputter-cleaned polycrystalline Ag foil, with the binding energy scale referenced both to the Ag $3d_{5/2}$ peak and the Fermi edge.

Table S4. Relative amounts of carbon, oxygen, fluorine and sulfur (in %) from the XPS survey spectra for the BDD control and Ar/SF_6 dry etching surfaces.

	**BDD control**		**Ar/SF_6 etched BDD**	
**Take-off angle 30°**	C	**98.1**	C	**93.6**
	O	**1.9**	O	**0**
	F	**0**	F	**4.5**
	S	**0**	S	**1.9**

The XPS C 1s and O 1s spectra for the BDD control are shown Figures S10a and b respectively. The C 1s spectrum is dominated by two peaks which can be attributed to sp^3 bonded carbon (85.6%) and sp^2 carbon (11.4%), respectively. The majority of the sp^2 carbon signal is likely to be due to sub-surface sp^2 carbon, which is generated during the mechanical polishing process.[22] Very small contributions from C-O-C (2.6%) and C=O (0.3%) are present. In Figure S9b, the O 1s spectrum shows two O species: the low energy binding peak is attributed to carbonyl groups (C=O), while the higher binding energy peak to ether groups (C−O−C),[14] as

expected for a (110) textured surface.[23, 24]

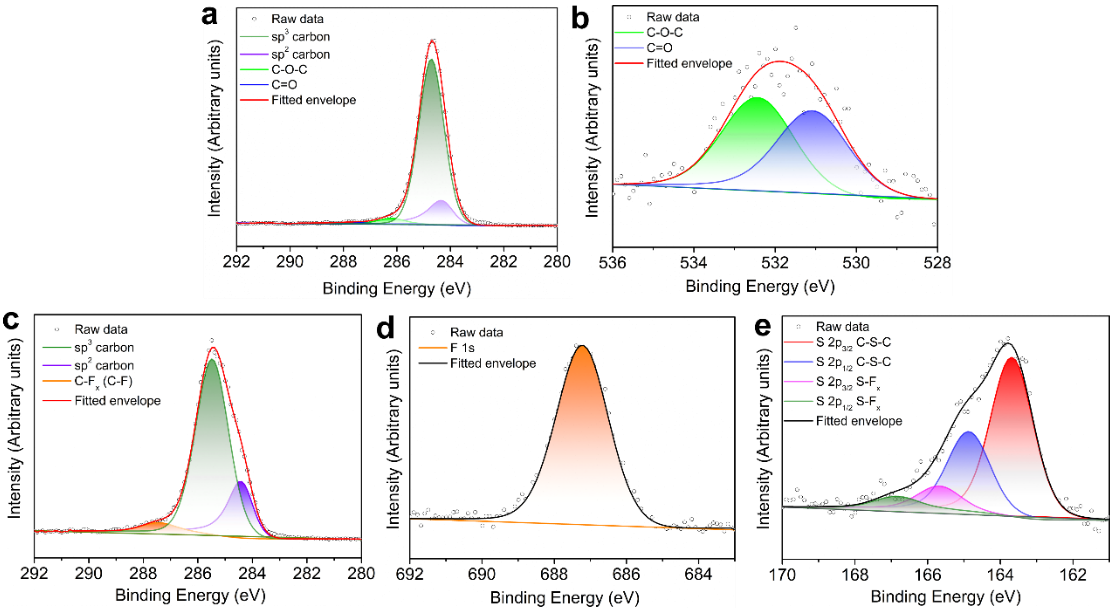

Figure S10. XPS spectra of the BDD control sample surface (a) carbon 1s (C 1s) spectrum. (b) Oxygen 1s (O 1s) spectrum and the Ar/SF_6 dry etching BDD surface (c) Carbon 1s (C 1s) spectrum. (d) Fluorine 1s (F 1s) spectrum. (e) Sulfur 2p (S 2p) spectrum. Spectra were taken at 30° take-off angle after annealing the sample to 500°C.

Table S5. C 1s fittings of the BDD oxygen terminated control and Ar/SF_6 dry etched surface expressed as percentages of the total fitted envelope.

	**BDD control**			**Ar/SF_6 dry etching treated BDD**		
	**Binding Energy (eV)**	**Relative composition (%)**		**Binding Energy (eV)**	**Relative composition (%)**	
**Take-off angle 30°**	284.71	sp^3 carbon	**85.6**	285.47	sp^3 carbon	**78.5**
	284.34	sp^2 carbon	**11.4**	284.43	sp^2 carbon	**17.8**
	286.22	C-O-C	**2.6**	287.45	C-F_x (C-F)	**3.8**
	287.69	C=O	**0.3**			

Table S6. O 1s fittings of the BDD oxygen terminated control expressed as percentages of the total fitted envelope.

	**O 1s fitting**		
	**Binding Energy (eV)**	**Relative composition (%)**	
**Take-off angle 30°**	532.41	C-O-C	**52.1**
	531.06	C=O	**47.9**

Table S7. F 1s and S 2p fittings of the Ar/SF_6 dry etched surface expressed as percentages of the total fitted envelope.

	**F 1s fitting**			**S 2p fitting**		
	**Binding Energy (eV)**	**Relative composition (%)**		**Binding Energy (eV)**	**Relative composition (%)**	
**Take-off angle 30°**	687.21	F 1s	**100**	163.68	S $2p_{3/2}$ C-S-C	**44.2**
				164.86	S $2p_{1/2}$ C-S-C	**43.3**
				165.68	S $2p_{3/2}$ S-F_x	**6.4**
				166.86	S $2p_{1/2}$ S-F_x	**6.2**

SI 6. Contact angle and electrochemical solvent window measurements

Contact angles were measured to investigate the hydrophobicity of the different BDD surfaces (Figure S11a). The average contact angles were 69.7 ± 2.2° and 63.8 ± 0.1° (n = 3) for the Ar/SF_6 treated BDD and the oxygen-terminated control BDD, respectively.

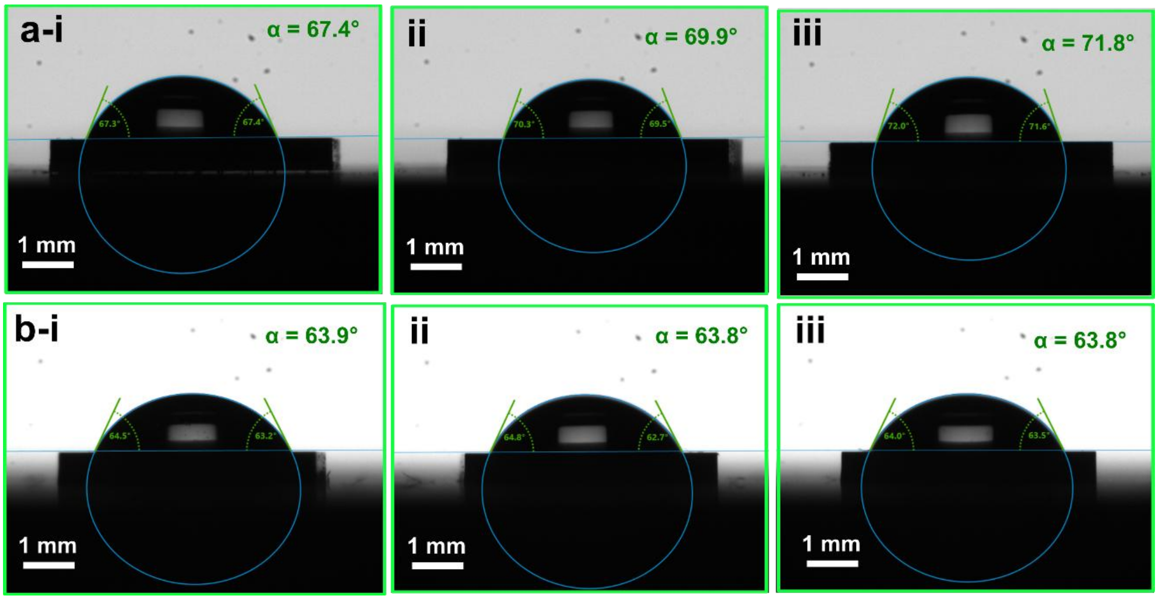

Figure S11a. Contact angles for a water droplet on (a) Ar/SF_6 treated BDD surface and (b) control oxygen-terminated BDD surface. The measurements were repeated three times to reduce errors.

An electrochemical solvent window in 0.1 M KNO_3 was recorded using the S/F terminated BDD (Ar/SF_6 treated), Figure S11b. The set-up comprised a three-electrode droplet cell with a 2 mm diameter disk area exposed using Kapton tape. Using a current density threshold of 0.4 mA cm^{-2}, the solvent window was determined as ~3.6 V.

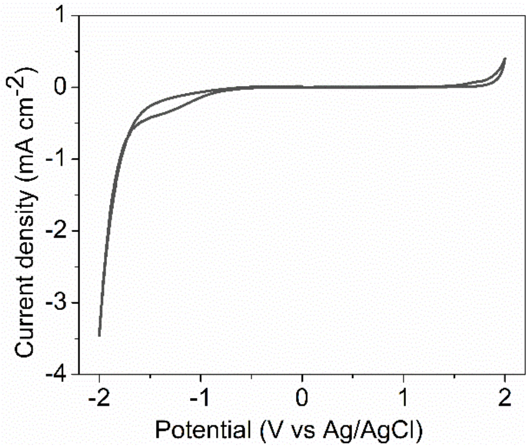

Figure S11b. First scan of solvent window of the S/F terminated BDD recorded at 100 mV s^{-1} in 0.1 M KNO_3.

SI 7. EELS analysis

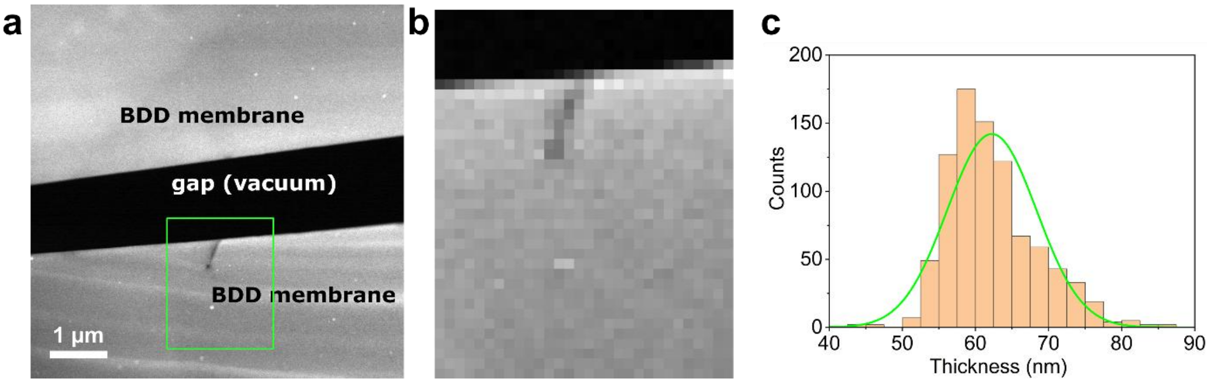

Figure S12. (a) ADF-STEM image of the BDD membrane. The gap is an area where a defective region has been completely etched through. The green square area was selected for EELS analysis. (b) The pixel image, acquired over the selected area of (a). (c) The thickness histogram profile extracted over the pixel image of (b), excluding vacuum. The mean thickness is 62 nm ± 6 nm (870 pixels).

SI 8. Optical measurements

SI 8a. Fourier Transform Infrared (FTIR) spectroscopy on BDD TEM membrane

To quantitatively measure the transmission of the BDD TEM membrane, FTIR was carried out with a Thermo Scientific Nicolet iN10 MX FTIR microscope with a spectral range of 800 (12.5 μm) to 7600 cm^{-1} (1.32 μm). A liquid nitrogen cooled MCT-A detector was used. The detector required 30 min for cooling and stabilization before data collection. Measurements, averaged over 256 scans, were performed on the BDD membrane in 50 × 50 μm regions, where the membrane either sat directly over a frame hole or a frame support,. As shown in Figure S13, when the membrane sits directly on the thick frame, transmission is ~0. However in the region of the membrane (~ 50 nm thickness), the transmission decreases as the wavenumber increases, plateauing at ~ 54% from 5500 -7500 cm^{-1}. For normal incidence of light at an air-diamond interface the reflection coefficient is

$$R = (n_\lambda - 1)^2/(n_\lambda + 1)^2 \qquad (1)$$

where n_λ is the refractive index at the wavelength, λ. At 1.5 μm, $n_\lambda = 2.39$ so $R = 0.17$ and when accounting for multiple reflections between each surface this leads to a maximum transmission of $(1 - R)^2/(1 - R^2) = 71\%$. The measured transmission of ~54% is only ~17% less than the theoretical maximum.

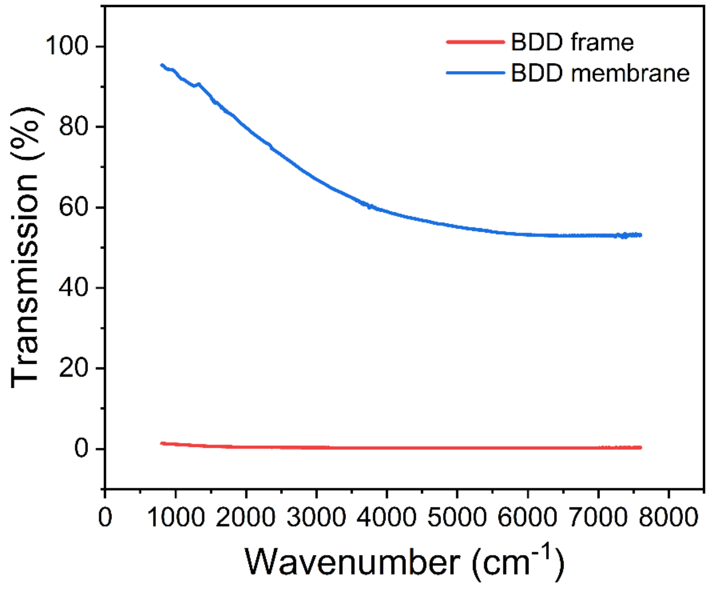

Figure S13. FTIR spectroscopy spectra of a BDD membrane, showing % transmission versus wavenumber in an area of the membrane versus an area of the support (frame).

SI 8b. Fluorescence Images of fluorescent amine-modified polystyrene particles on the BDD membrane

A fluorescent particle (bead) solution was prepared by diluting 5 µL of a concentrated amine-modified polystyrene particles suspension (fluorescent yellow-green, approximate excitation/emission of 488/509 nm, 1.0 µm mean particle size, aqueous suspension, Sigma) were added into 25 mL of water. A single-barreled SECCM micropipet probe (~40 µm in diameter) was filled with the solution. The probe was approached to the BDD membrane surface using an inverted optical microscope-coupled SECCM workstation.[25] The particles were drop casted onto the membrane by bringing the solution meniscus, hanging from the micropipet probe, in contact with the surface and then retracting the probe. The landing site was observed under 100× optical magnification (63× oil immersion microscope objective lens, HCX Plan Apochromatic, numerical aperture equal to 1.4, Leica, Germany; and further 1.6× magnification by the microscope's tube lens, DMI4000B inverted microscope, Leica). Illumination was supplied by: (i) an Aura Phase Contrast Illuminator white LED light source (Cairn Research, UK), for recording transmitted light images; or (ii) a blue LED from a Niji light source (peak intensity wavelength of 470 nm, Bluebox Optics, UK), for recording reflected light fluorescence images. For the fluorescence recordings, an appropriate filter cube was used (I3, excitation filter 450–490 nm BP, suppression filter 515 nm LP, Leica). Optical images were recorded using a CMOS digital camera (C11440-42U30, Hamamatsu Photonics, Japan).

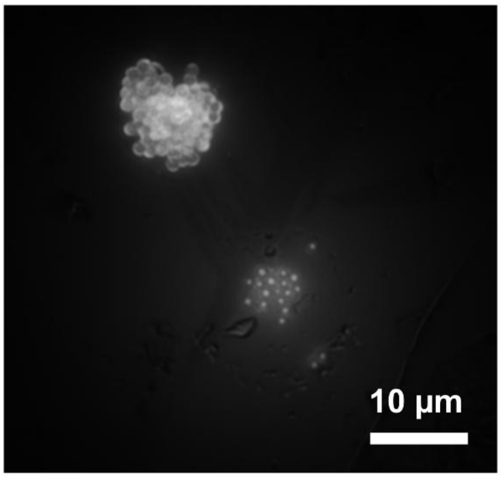

Figure S14. Fluorescence image of fluorescent amine-modified polystyrene particles on the BDD membrane (reflection mode, inverted microscope).

SI 9. SECCM setup and electrochemical measurements

9.1 Scanning Electrochemical Probe Microscopy (SEPM) workstation

The SEPM workstation consisted of *x-y* and *z* piezoelectric positioners (respectively P-733.2DD and P-753.11C, Physik Instrumente, Germany) for fine position control; *x,y,z* coarse micropositioners (M-461-XYZ-M, Newport, U.S.A.) for initial positioning; a home-built electrometer featuring current amplification with a sensitivity of 10 pA/V; and a data collection instrument control system (PCIe-7852R FPGA card, National Instruments, U.S.A.) in a PC workstation.[26] The positioning of the sample and the probe with this equipment was aided by an optical camera (PLB776U camera equipped with a 8× lens, Pixelink, U.S.A.). The SECCM instrument was placed on a passive mechanical vibration isolator platform (Minus K Technology, U.S.A.) housed in an aluminium Faraday cage, equipped with heat sinks and acoustic foam to minimize disturbances from mechanical, electromagnetic, thermal, or acoustic sources.

A single-barreled nanopipette probe (~ 200 nm) was filled with electrolyte solution for gold electrodeposition (20 μM $HAuCl_4$) forming an electrolyte solution meniscus at the tip. A palladium (Pd) wire was inserted at the other end of the probe, positioned ca. 3 cm from the tip end and used as quasi-reference-counter electrode (QRCE). The probe was lowered into position near the sample surface using the coarse micropositioners (ca. 20 μm above the surface) assisted by the optical camera. The nanopipette was then approached to the sample while continuously monitoring (via the electrometer) the current collected at the BDD membrane, connected as the working electrode. This was used as a feedback signal, and nanopipette descent was stopped automatically by the instrument when a threshold current value was reached, signaling contact of the meniscus with the electrode surface. For CV measurements, during probe approach, the applied potential was +0.8 V *vs* Pd/Pd-H_2 (threshold current = 0.55 pA), ensuring that gold deposition did not occur upon landing. The rate of probe approach was 2 μm s^{-1}. Once the meniscus had made contact, a CV in the potential range +1.8 to - 0.3 V *vs* Pd/Pd-H_2, was immediately commenced, at a scan rate of 0.5 V s^{-1}. For chronoamperometry measurements, the capillary was approached at +1.5 V *vs* Pd/Pd-H_2 where no deposition occurs and upon contact immediately switched to different cathodic potentials (i) 0.20 V, (ii) 0.00 V and (iii) - 0.20 V *vs* Pd/Pd-H_2, for 0.5 s. The probe was then retracted at a potential of +0.8 V *vs* Pd/Pd-H_2 at a speed of 10 μm s^{-1} and moved to a new location.

All data acquisition and instrumental control was carried out using the FPGA card, controlled

by a LabVIEW 2019 (National Instruments) interface, running the Warwick Electrochemical Scanning Probe Microscopy (WEC-SPM, www.warwick.ac.uk/electrochemistry) software. The SECCM data were processed using custom scripts running on the Matlab R2021b (Mathworks, U.S.A.) software suite and plotted with OriginPro 2021 64bit (9.60, OriginLab, U.S.A.) software.

9.2 Probe fabrication

The single-barreled nanopipette probes were fabricated by pulling borosilicate filamented capillaries (GC120F-10, Harvard Apparatus, U.S.A.) to a sharp point using a CO_2-laser puller (P-2000, Sutter Instruments, U.S.A.), employing the following pulling parameters:

HEAT 385, FIL 3, VEL 30, DEL 210, PUL -

HEAT 385, FIL 3, VEL 40, DEL 170, PUL 120

An SEM image of a typical nanopipette tip is shown in Figure S15. The tip diameter was ~ 200 nm.

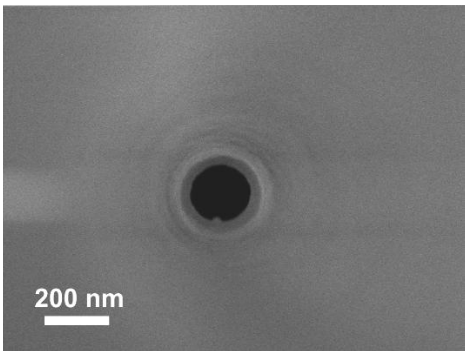

Figure S15. SEM image of the SECCM tip (end-on)

9.3 Electrodes

Pd/Pd-H_2 QRCEs were prepared by applying a potential difference of -3.0 V for 30 min to Pd wire in 0.1 M H_2SO_4 using a platinum mesh as the counter electrode. Prior to use, the potential of this QRCE was calibrated by measuring its open circuit potential in the employed solution with respect to a commercial 3.4 M Ag/AgCl leak free reference electrode (ET072, eDAQ, Australia). The latter has a theoretical standard potential of + 205 mV *vs* the standard hydrogen electrode.[27] The Pd/Pd-H_2 QRCE possessed a stable potential of -206 mV vs. Ag/AgCl. All the potentials of the SECCM experiments reported in this work are referred to versus the Pd/Pd-H_2 electrode.

The BDD TEM membrane was mounted on a 2.5 cm SEM stub, equipped with two Cu pins (AGG3032-252, Agar Scientific, U.K.) to immobilize the sample and make electrical contact.

9.4 Electrochemical measurements

For CV measurements, three complete CVs were recorded with a sampling time of 4 μs, averaged 256 times. For chronoamperometry measurements, six deposition lines were made on the BDD TEM electrode for three deposition potentials (two lines for each potential). Each line contains ten gold depositions and the distance between each point was set to 3 μm. The data were recorded with a sampling time of 4 μs averaged 32 times. For the deposition times, given the solution conditions (no supporting electrolyte) and tip sizes employed, the current-time responses were dominated by charging (R_uC) and so no further analysis of these data were made.

The average NP diameters were estimated from the ADF-STEM images in Figure S16 using ImageJ software, for particles ≥ 7.9 nm in diameter (based on image resolution in Figure S16). First, the NP area, A, was manually selected using a threshold and then the diameter (2r where r = radius) calculated based on the equation of $A=\pi r^2$ (with the assumption of circular particles). The results are summarized in Table S8.

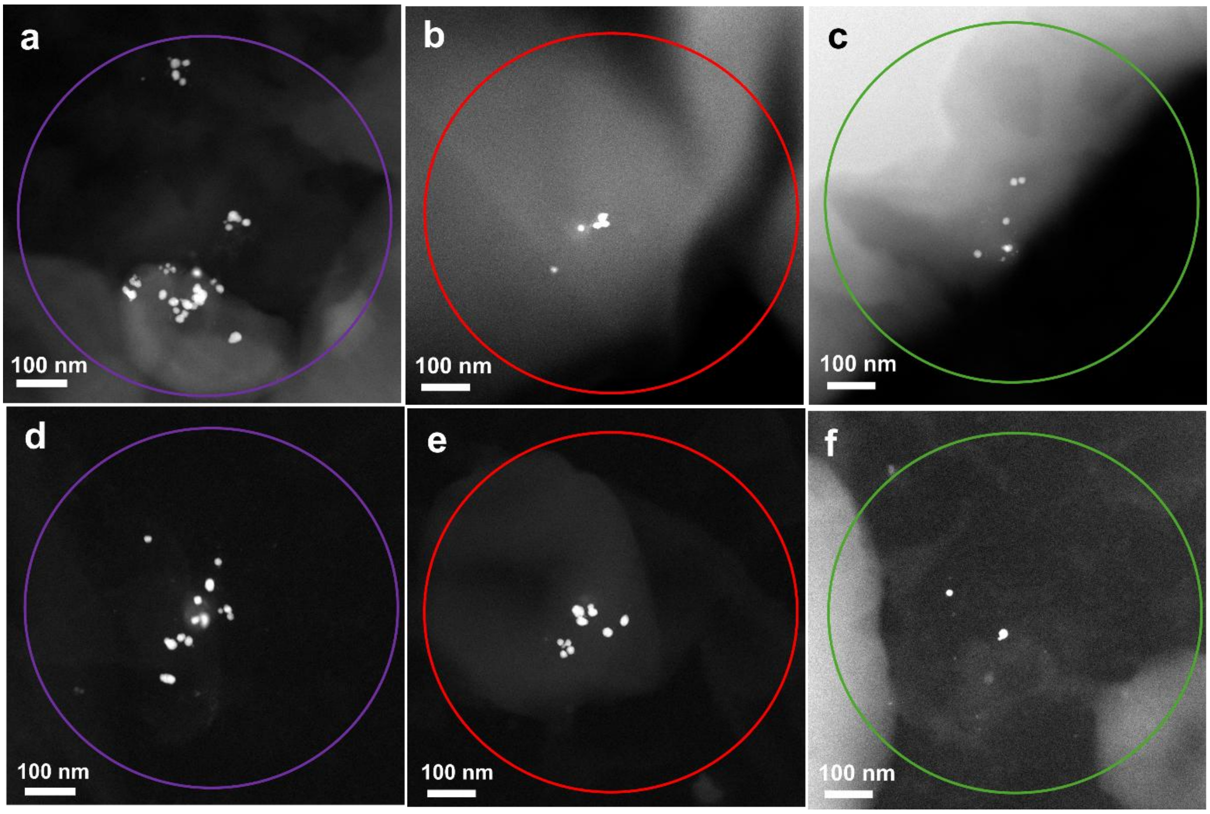

Figure S16. Low magnification ADF-STEM images of electrodeposited gold on the BDD membrane

at -0.20 V (a, d), 0.00 V (b, e) and 0.20 V *vs* Pd/Pd-H_2 (c, f) for 0.5 s, showing six spatially-independent SECCM footprint regions which corresponds to the six meniscus footprints in the orange rectangle area in Figure 4b. The footprints for the different potentials are indicated by the purple (-0.20 V), red (0.00 V) and green (+0.20 V) circles. Note that the circles represent only approximate locations of the SECCM meniscus footprints.

Table S8. Summary of the average particle size within the low resolution TEM image of the six droplets in Figure S16 (from top to bottom).

Deposited potential (V)	Average particle diameter from TEM image (nm)
-0.20	20.8 ± 10.9
0.00	18.2 ± 7.1
0.20	13.8 ± 2.1

Table S9. Statistical analysis of single crystal gold NPs electrodeposited at different potentials (for NPs with a diameter bigger than 7 nm in the six meniscus footprints).

Deposited potential	-0.20 V	0.00 V	0.20 V
**NP numbers**	45	16	8
**Single crystal NPs**	0	1	3
**Percentage of single crystal NPs (%)**	0	6%	38%

SI 10. Details on the different measurements made for assessment of the BDD membrane versus C thin film

10.1 Mechanical strength test

In order to obtain a qualitative comparison of mechanical strength, force-distance (F-d) approach-retract curves were conducted using the AFM operating in the Mechanical Properties mode. A very thick film of sapphire was used as the reference hard material for calibration of the probe spring constant (31.5 N/m). The resulting approach-retract F-d data is shown by the black/red line data in Figure S17, where there is no deformation of the sample. Repeating this measurement against the BDD membrane (green/black) and the carbon (C) film (blue/purple) resulted in the F-d data shown in Figure S17 (data offset to give the same point of contact with the surface). In both cases the gradient is less due to the sample deforming as well as the cantilever; the smaller the gradient the greater the deformation. Sample deformation is most severe for the C film.

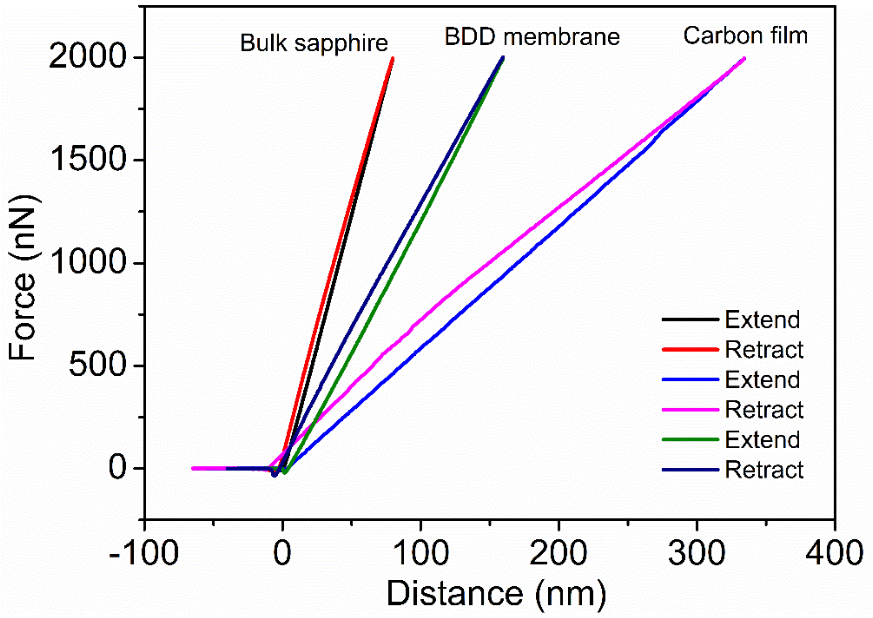

Figure S17. Force-distance curves (approach and retract) for the three materials: Bulk sapphire, BDD membrane and C film.

10.2 Ex situ heating IL-TEM test

The *ex situ* heating test was performed at 500 °C in air for 4 h, at a heating rate of 10 °C min^{-1}. IL-TEM images of the C film (Cu) TEM grid and BDD membrane were recorded before and after heating using a JEOL JEM 2100 TEM at 200 kV. After heating, the C has disappeared, due to thermal oxidation and a thorny Cu mesh left behind (compare Figure S18a with S18b).

The square shaped hole in the Cu grid has also shrunk. Such features are most likely due to Cu to Cu oxides phase transformations and heat induced surface mobility of Cu atoms. The BDD membrane retains intact during heating and appears cleaner. For *ex-situ* heating experiments the BDD membrane offers thermal stability advantages over the C film / Cu TEM grid.

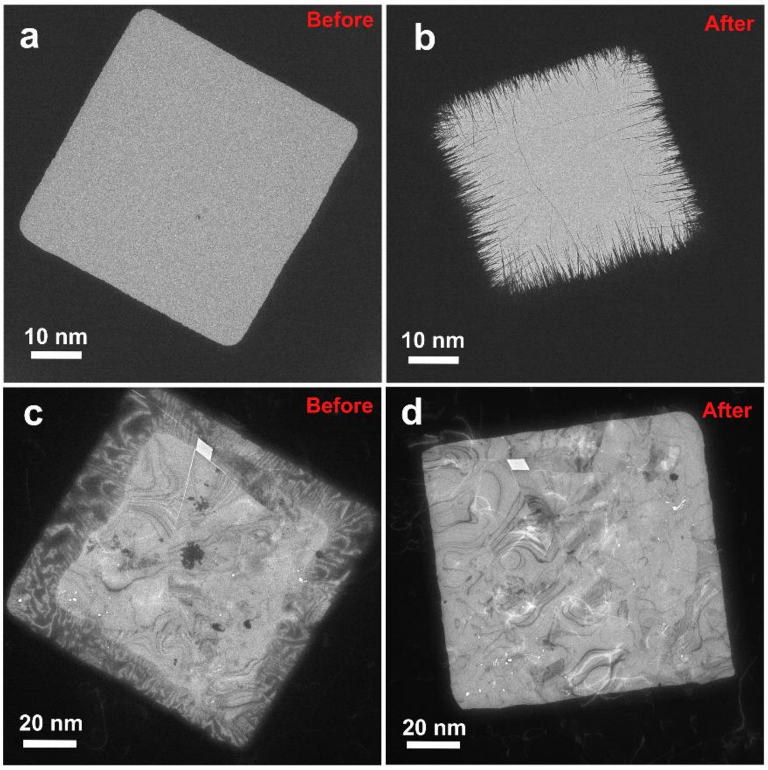

Figure S18. Low magnification bright field IL-TEM image of one square region of (a, b) the C film / Cu TEM grid and (c, d) BDD membrane (on the BDD support), imaged at 200 kV. *Ex-situ* heating was performed at 500 °C in air for 4 h.

10.3 High resolution imaging under in situ heating conditions at 400°C

This measurement was carried out in a double aberration-corrected JEOL JEM-ARM 200F TEM operated at 200 kV in conjunction with an *in-situ* heater. For both the BDD membrane and the C film a relatively high intensity electron beam (current ~2 nA) was focused on a small area (~40 nm diameter, giving a current density at the sample of ~1.6×10^6 A/m^2) at a nominal temperature of 400 °C. At 30 s intervals the beam was defocused to illuminate a large area and a TEM image was recorded. Damage was sufficiently rapid that an illumination time of 90 s was sufficient to completely alter the amorphous C support film. In fact, as shown in Figure S19a-c, graphitisation and thinning of the amorphous C is apparent even after 30 s. In contrast, there is no obvious change for the BDD

membrane, demonstrating its high stability under high current density (as used in high resolution imaging) and at elevated temperature. The diffuse dark lines in d-f are diffraction contours, a consequence of using a slightly convergent electron beam for illumination.

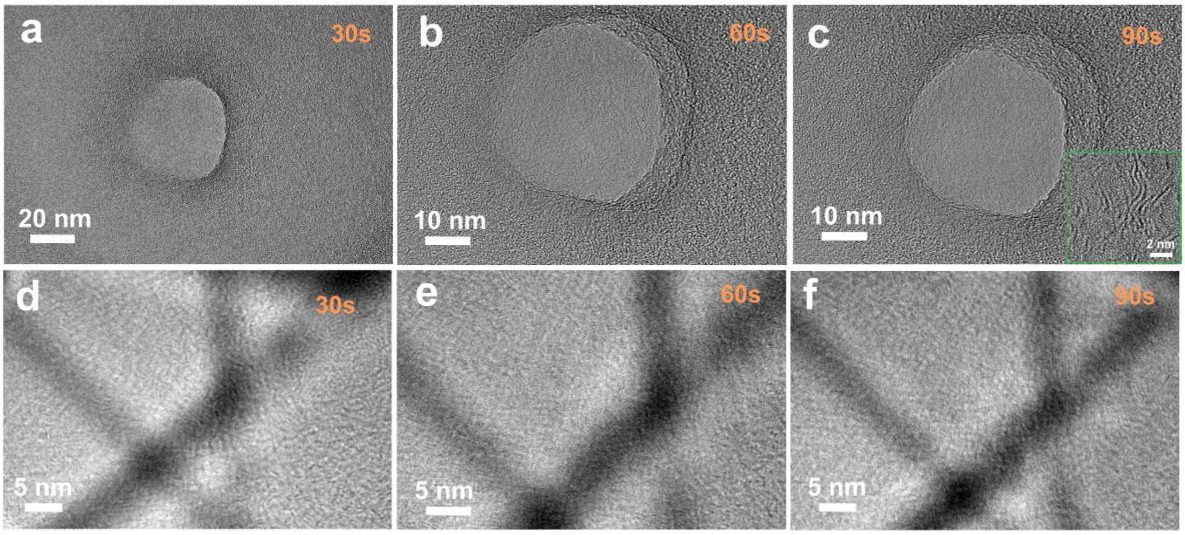

Figure S19. ADF-STEM images of (a-c) C film and (d-f) BDD membrane during the *in-situ* heating, high intensity electron beam test. The current density is ~1.6×10^6 A/m^2 and the temperature is 400 °C. Inset to (c) shows initial stages of C film graphitization.

10.4 SECCM on C film (Cu) TEM grid

Repetitive CV cycles from 0.8 to 2.0 V *vs* Pd/Pd-H_2 were conducted using a pipette tip of diameter ~ 500 nm at 0.25 V s^{-1} in 0.5 M H_2SO_4. Figure S20a shows the SEM image of four footprints (#1~4) on the C film (Cu) TEM grid after SECCM CV cycling. The upper potential is sufficient to initiate carbon corrosion for sp^2 bonded C.[28] A large hole with cracks in the film was observed to originate from SECCM footprint #1 after 6 CV cycles, Figure S20b. The current increases rapidly with increasing scan number most likely due to hole formation. Much smaller cracks (or corrosion features) in the C film are also found associated with SECCM footprints #2-4 (Figure S20c-e). Here the SECCM currents decrease with increasing cycle number (Figure S20c-e). The response to the SECCM cycling in acid does vary across the SECCM footprints also indicating possible variations in the material properties of the film. The fragility of the C film is also evident in Figure S20f. Even careful handing with tweezers results in damage to the C film and loss of usable area. In contrast no obvious changes to the BDD surface were observed using SEM and handling of the film did not result in damage.

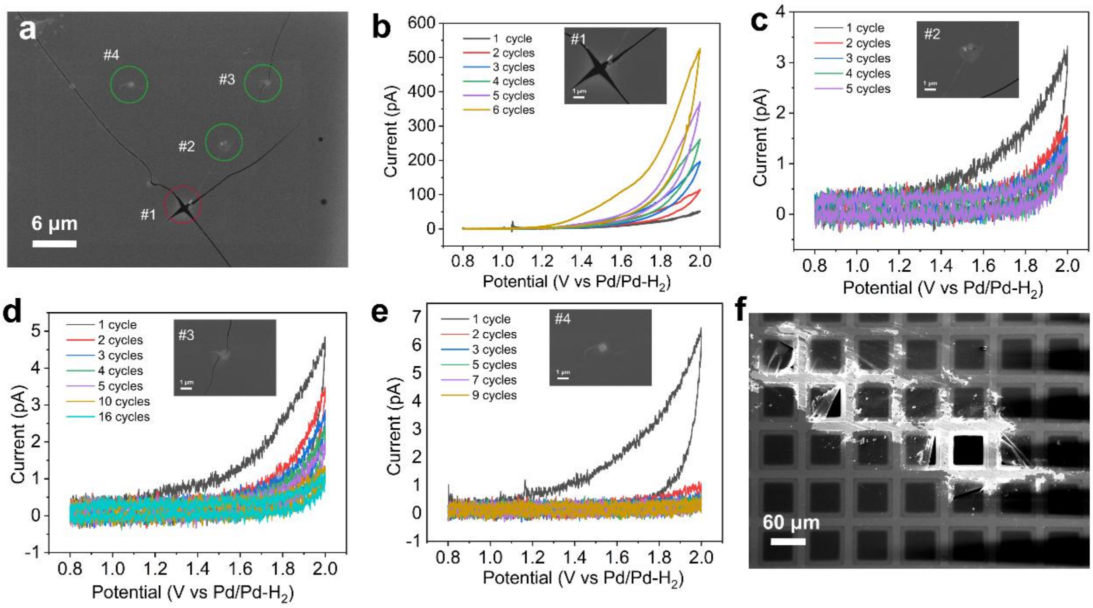

Figure S20. (a) SEM image of the four SECCM meniscus footprints on the C film (Cu) TEM grid. The four footprint areas are indicated using circles #1, #2, #3 and #4. The hole and cracks on the C film (Cu) TEM grid are evident. (b-e) Repetitive CVs on the C film (Cu) TEM grid using SECCM at a scan rate of 0.25 V s^{-1}. The insets are the four footprint areas in (a). The electrolyte was 0.5 M H_2SO_4 and the nanopipette diameter was ~ 500 nm. (f) SEM image of the damaged C film (Cu) TEM grid, showing its fragility even after careful handling during the SECCM experiment.

SI 11. Electrochemical etching set-up for membrane lift-off

The electrochemical etching process took place in a custom designed 3D printed (Form 3, FormLabs, USA), made from PMMA (Form Clear), as shown in Figure S21. The 3D cell model can be downloaded from https://www.printables.com/model/1349319. A variable DC power supply (EA-PS 9750-04, Elektro-Automatik GmbH, Germany) was employed to apply a 30 V potential between two Pt wire electrodes (0.75 mm in diameter and 2 cm in length) resulting in a current of ca. 0.1 - 0.2 A. A home-built device was used to switch the polarity every 60 s, so that each Pt electrode spent half the time as the anode. The ion implanted BDD substrate was attached to a polycarbonate (RS Components, UK) support using UV resin, which itself was placed into a slot between the two Pt electrodes (6 mm space apart). Another piece of polycarbonate was also inserted to make sure the sp^2 carbon layer was held in plane with the Pt wire electrodes and the face of the BDD was perpendicular to the base of the etch cell.

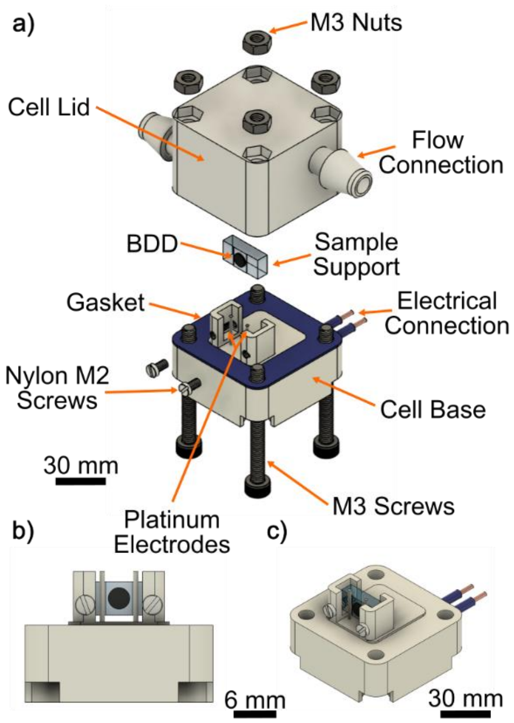

Figure S21. Rendering of the cell design (a) overlook view of different components. (b) Front view. (c) Side view.

A flow system with a temperature-controlled reservoir operating at 25°C was used to keep the solution composition and temperature constant.[2] Etch solution (0.05 M H_2SO_4) was circulated between the reservoir and the cell via a pump at a flow rate of 200 mL/min^{-1}. To minimize the effect of solution flow on the BDD sample and Pt electrodes, the inlet and outlet were placed

far away from electrodes. The sp^2 carbon layer was removed after 10 – 12 hours of etching depending on the sample. A video of the etching process is available for download.

References

1. Ziegler, J. F.; Ziegler, M. D.; Biersack, J. P., SRIM – The stopping and range of ions in matter (2010). *Nuclear Instruments and Methods in Physics Research Section B: Beam Interactions with Materials and Atoms* **2010,** *268* (11), 1818-1823.
2. Tully, J. J.; Braxton, E.; Cobb, S. J.; Breeze, B. G.; Markham, M.; Newton, M. E.; Rodriguez, P.; Macpherson, J. V., Diamond membrane production: The critical role of radicals in the non-contact electrochemical etching of sp2 carbon. *Carbon* **2021,** *185*, 717-726.
3. Uzan-Saguy, C.; Cytermann, C.; Brener, R.; Richter, V.; Shaanan, M.; Kalish, R., Damage threshold for ion-beam induced graphitization of diamond. *Applied Physics Letters* **1995,** *67* (9), 1194-1196.
4. Bosia, F.; Argiolas, N.; Bazzan, M.; Olivero, P.; Picollo, F.; Sordini, A.; Vannoni, M.; Vittone, E., Modification of the structure of diamond with MeV ion implantation. *Diamond and Related Materials* **2011,** *20* (5), 774-778.
5. Olivero, P.; Rubanov, S.; Reichart, P.; Gibson, B. C.; Huntington, S. T.; Rabeau, J. R.; Greentree, A. D.; Salzman, J.; Moore, D.; Jamieson, D. N.; Prawer, S., Characterization of three-dimensional microstructures in single-crystal diamond. *Diamond and Related Materials* **2006,** *15* (10), 1614-1621.
6. Prins, J. F., Ion-implanted structures and doped layers in diamond. *Materials Science Reports* **1992,** *7* (7), 275-364.
7. Bosia, F.; Calusi, S.; Giuntini, L.; Lagomarsino, S.; Lo Giudice, A.; Massi, M.; Olivero, P.; Picollo, F.; Sciortino, S.; Sordini, A.; Vannoni, M.; Vittone, E., Finite element analysis of ion-implanted diamond surface swelling. *Nuclear Instruments and Methods in Physics Research Section B: Beam Interactions with Materials and Atoms* **2010,** *268* (19), 2991-2995.
8. Fairchild, B. A.; Rubanov, S.; Lau, D. W. M.; Robinson, M.; Suarez-Martinez, I.; Marks, N.; Greentree, A. D.; McCulloch, D.; Prawer, S., Mechanism for the Amorphisation of Diamond. *Advanced Materials* **2012,** *24* (15), 2024-2029.
9. Ruf, M.; Ijspeert, M.; van Dam, S.; de Jong, N.; van den Berg, H.; Evers, G.; Hanson, R., Optically Coherent Nitrogen-Vacancy Centers in Micrometer-Thin Etched Diamond Membranes. *Nano Letters* **2019,** *19* (6), 3987-3992.
10. Guo, X.; Delegan, N.; Karsch, J. C.; Li, Z.; Liu, T.; Shreiner, R.; Butcher, A.; Awschalom, D. D.; Heremans, F. J.; High, A. A., Tunable and Transferable Diamond Membranes for Integrated Quantum Technologies. *Nano Letters* **2021,** *21* (24), 10392-10399.
11. Hausmann, B. J. M.; Khan, M.; Zhang, Y.; Babinec, T. M.; Martinick, K.; McCutcheon, M.; Hemmer, P. R.; Lončar, M., Fabrication of diamond nanowires for quantum information processing applications. *Diamond and Related Materials* **2010,** *19* (5), 621-629.
12. Challier, M.; Sonusen, S.; Barfuss, A.; Rohner, D.; Riedel, D.; Koelbl, J.; Ganzhorn, M.; Appel, P.; Maletinsky, P.; Neu, E. Advanced Fabrication of Single-Crystal Diamond Membranes for Quantum Technologies *Micromachines* [Online], 2018.
13. Wilson, N. R.; Clewes, S. L.; Newton, M. E.; Unwin, P. R.; Macpherson, J. V., Impact of Grain-Dependent Boron Uptake on the Electrochemical and Electrical Properties of Polycrystalline Boron Doped Diamond Electrodes. *The Journal of Physical Chemistry B* **2006,** *110* (11), 5639-5646.

14. Chaudhuri, S.; Hall, S. J.; Klein, B. P.; Walker, M.; Logsdail, A. J.; Macpherson, J. V.; Maurer, R. J., Coexistence of carbonyl and ether groups on oxygen-terminated (110)-oriented diamond surfaces. *Communications Materials* **2022,** *3* (1), 6.
15. Macpherson, J. V., A practical guide to using boron doped diamond in electrochemical research. *Physical Chemistry Chemical Physics* **2015,** *17* (5), 2935-2949.
16. Liu, Z.; Baluchová, S.; Sartori, A. F.; Li, Z.; Gonzalez-Garcia, Y.; Schreck, M.; Buijnsters, J. G., Heavily boron-doped diamond grown on scalable heteroepitaxial quasi-substrates: A promising single crystal material for electrochemical sensing applications. *Carbon* **2023,** *201*, 1229-1240.
17. Yang, G.; Lu, Y.; Wang, B.; Xia, Y.; Chen, H.; Song, H.; Yi, J.; Deng, L.; Wang, Y.; Li, H. Chemical Vapor Deposition of <110> Textured Diamond Film through Pre-Seeding by Diamond Nano-Sheets *Materials*, 2022, *15* (21), 7776-7782.
18. Tougaard, S., QUASES-IMFP-TPP2M software, version 3.0, available from http://www.quases.com.
19. Gunter, P. L. J.; De Jong, A. M.; Niemantsverdriet, J. W.; Rheiter, H. J. H., Evaluation of take-off-angle-dependent XPS for determining the thickness of passivation layers on aluminium and silicon. *Surface and Interface Analysis* **1992,** *19* (1-12), 161-164.
20. Tanuma, S.; Powell, C. J.; Penn, D. R., Calculation of electron inelastic mean free paths (IMFPs) VII. Reliability of the TPP-2M IMFP predictive equation. *Surface and Interface Analysis* **2003,** *35* (3), 268-275.
21. Bobrov, K.; Shechter, H.; Hoffman, A.; Folman, M., Molecular oxygen adsorption and desorption from single crystal diamond (1 1 1) and (1 1 0) surfaces. *Applied Surface Science* **2002,** *196* (1), 173-180.
22. Zheng, Y.; Ye, H.; Thornton, R.; Knott, T.; Ochalski, T. J.; Wang, J.; Liu, J.; Wei, J.; Chen, L.; Cumont, A.; Zhang, R.; Li, C., Subsurface cleavage of diamond after high-speed three-dimensional dynamic friction polishing. *Diamond and Related Materials* **2020,** *101*, 107600.
23. Hussein, H. E. M.; Wood, G.; Houghton, D.; Walker, M.; Han, Y.; Zhao, P.; Beanland, R.; Macpherson, J. V., Electron Beam Transparent Boron Doped Diamond Electrodes for Combined Electrochemistry—Transmission Ele.ctron Microscopy. *ACS Measurement Science Au* **2022,** *2* (5), 439-448.
24. Hussein, H. E. M.; Maurer, R. J.; Amari, H.; Peters, J. J. P.; Meng, L.; Beanland, R.; Newton, M. E.; Macpherson, J. V., Tracking Metal Electrodeposition Dynamics from Nucleation and Growth of a Single Atom to a Crystalline Nanoparticle. *ACS Nano* **2018,** *12* (7), 7388-7396.
25. Valavanis, D.; Ciocci, P.; Meloni, Gabriel N.; Morris, P.; Lemineur, J.-F.; McPherson, I. J.; Kanoufi, F.; Unwin, P. R., Hybrid scanning electrochemical cell microscopy-interference reflection microscopy (SECCM-IRM): tracking phase formation on surfaces in small volumes. *Faraday Discussions* **2022,** *233* (0), 122-148.
26. Ebejer, N.; Güell, A. G.; Lai, S. C. S.; McKelvey, K.; Snowden, M. E.; Unwin, P. R., Scanning Electrochemical Cell Microscopy: A Versatile Technique for Nanoscale Electrochemistry and Functional Imaging. *Annual Review of Analytical Chemistry* **2013,** *6* (1), 329-351.
27. Spitzer, P.; Wunderli, S.; Maksymiuk, K.; Michalska, A.; Kisiel, A.; Galus, Z.; Tauber, G., Reference Electrodes for Aqueous Solutions. In *Handbook of Reference Electrodes*, Inzelt, G.; Lewenstam, A.; Scholz, F., Eds. Springer Berlin Heidelberg: Berlin, Heidelberg, 2013; pp 77-143.
28. Yi, Y.; Weinberg, G.; Prenzel, M.; Greiner, M.; Heumann, S.; Becker, S.; Schlögl, R., Electrochemical corrosion of a glassy carbon electrode. *Catalysis Today* **2017,** *295*, 32-40.
